# Identification and characterization of putative *Aeromonas* spp. T3SS effectors

**DOI:** 10.1371/journal.pone.0214035

**Published:** 2019-06-04

**Authors:** Luiz Thiberio Rangel, Jeremiah Marden, Sophie Colston, João Carlos Setubal, Joerg Graf, Johann Peter Gogarten

**Affiliations:** 1 Department of Molecular and Cell Biology, University of Connecticut, Storrs, Connecticut, United States of America; 2 Interunidades em Bioinformática, Universidade de São Paulo, São Paulo, Brasil; 3 Departamento de Bioquímica, Instituto de Química, Universidade de São Paulo, São Paulo, Brasil; 4 Institute for Systems Genomics, University of Connecticut, Storrs, Connecticut, United States of America; Oswaldo Cruz Foundation, BRAZIL

## Abstract

The genetic determinants of bacterial pathogenicity are highly variable between species and strains. However, a factor that is commonly associated with virulent Gram-negative bacteria, including many *Aeromonas* spp., is the type 3 secretion system (T3SS), which is used to inject effector proteins into target eukaryotic cells. In this study, we developed a bioinformatics pipeline to identify T3SS effector proteins, applied this approach to the genomes of 105 *Aeromonas* strains isolated from environmental, mutualistic, or pathogenic contexts and evaluated the cytotoxicity of the identified effectors through their heterologous expression in yeast. The developed pipeline uses a two-step approach, where candidate *Aeromonas* gene families are initially selected using Hidden Markov Model (HMM) profile searches against the Virulence Factors DataBase (VFDB), followed by strict comparisons against positive and negative control datasets, greatly reducing the number of false positives. This approach identified 21 *Aeromonas* T3SS likely effector families, of which 8 represent known or characterized effectors, while the remaining 13 have not previously been described in *Aeromonas*. We experimentally validated our *in silico* findings by assessing the cytotoxicity of representative effectors in *Saccharomyces cerevisiae* BY4741, with 15 out of 21 assayed proteins eliciting a cytotoxic effect in yeast. The results of this study demonstrate the utility of our approach, combining a novel *in silico* search method with *in vivo* experimental validation, and will be useful in future research aimed at identifying and authenticating bacterial effector proteins from other genera.

## Introduction

*Aeromonas* spp. are Gram-negative γ-proteobacteria, many of which are of increasing clinical significance as emergent human pathogens [1,2]. *Aeromonads* are present in diverse habitats and interact with a variety of organisms, such as leeches, zebrafish, vultures, and humans, participating in both mutualistic and pathogenic symbiotic interactions with their hosts [3–8]. *Aeromonas* species cause diseases in a variety of animals, and they are particularly associated with fish diseases caused by the psychrophilic species *A*. *salmonicida* as well as by mesophilic ones, such as *A*. *hydrophila*, *A*. *sobria* and *A*. *veronii* [5,9]. In humans, *Aeromonas* strains are identified as causative agents of traveler’s diarrhea and septicemia, as well as other severe infections such as necrotizing fasciitis [1,10].

Numerous important *Aeromonas* virulence factors have been identified, including aerolysin, exotoxins, and type three secretion systems (T3SSs) [11]. The T3SS is an important and one of the best-studied bacterial virulence factors [12–14], and the diversity of niches inhabited by *Aeromonas* spp. may be due in part to many strains possessing one or more T3SSs [15–21]. The T3SS is a molecular syringe that transfers effectors with a range of biochemical activities into target eukaryotic cells [22]. In addition, T3SSs are frequently associated with horizontal gene transfer (HGT) events and are commonly observed within pathogenicity islands [23–25]. Thus, the importance of T3SSs in bacterial-eukaryotic interactions and their frequent horizontal transfer makes them important players in niche adaptation processes [26].

Next-generation sequencing has driven remarkable advances in microbiology, both in fundamental research and clinical diagnostics [27,28]. A major objective in the acquisition and analysis of whole genome sequence data is to better predict the pathogenic potential of bacterial strains by identifying genetic determinants of virulence. The core components of the T3SS apparatus are homologous to the bacterial flagellum, and their sequences are sufficiently similar that cross identifications occur during homology searches. In contrast, T3SS effectors are less conserved and often display uncharacterized domains, which also leads to difficulties during homology searches. An example of these challenges are two effectors, AexT and AexU, in *Aeromonas*, which share one domain with a second domain being completely novel [29,30]. Databases such as the Pathosystems Resource Integration Center (PATRIC) [31], VICTORS [32], EffectiveDB [33], and the Virulence Factor DataBase (VFDB) [34] contain T3SS effector sequences with different levels of validation. One problem encountered when attempting to identify T3SS components through homology searches is that many proteins and protein domains encoded in bacterial genomes are homologs of T3SS components that are not part of a T3SS system, but rather encode other cellular components such as bacterial flagellar components [35], F-ATPase subunits [36], and type IV pili [37,38]. In this study, we describe an *in silico* approach that solves this problem through the use a negative control comprised of all protein sequences from *Vibrio fisherii* ES114 and *Escherichia coli* K12. Since these genomes are known to not encode a T3SS, their protein sequences can be used as non-T3SS references.

In this study, we report the distribution and cytotoxicity of 21 candidate T3SS effector families spanning 23 identified groups of homologs within 105 *Aeromonas* genomes and describe the pipeline developed to identify them. The identified proteins were evaluated as potential T3SS effectors by expressing representative proteins in the yeast *Saccharomyces cerevisiae* strain BY4741 and assessing their cytotoxicity. Out of 21 identified candidate effector families, 13 are newly described and 15 exhibited cytotoxicity under the conditions assayed. Our findings extend the knowledge of the breadth and distribution of T3SS effectors in *Aeromonas* strains, and our combined use of a bioinformatics pipeline followed by verification through heterologous expression in yeast provides a template for studies in other bacterial genera.

## Materials and methods

### Sequences

All annotated protein coding gene sequences from the 105 *Aeromonas* strains were assessed in this study. From the 105 evaluated *Aeromonas* genomes, 40 genomes were sequenced during this study (S1 Table), 35 were previously published by our group [21,39,40], and 30 were obtained from public databases (S2 Table). The genomes were sequenced, assembled and annotated as described in Colston et al. 2014 [40]. Briefly, libraries were prepared using NexteraXT and sequenced on an Illumina MiSeq at the Microbial Analysis, Resources and Services Facility of the University of Connecticut. The reads were trimmed and assembled using CLC Genomics Workbench (Qiagen). Gene predictions and product annotations were performed using RAST [41]. The data is available under BioProject PRJNA391781 and SRA accession numbers SRS2335044-SRS2335083.

### Homology clustering

Protein sequences from the 105 *Aeromonas* genomes were clustered into homologous groups using the OrthoMCL algorithm [42] as implemented in the Get_Homologues software package using default parameters [43]. A total of 25,518 homologous groups were assembled, 2,755 of which are present in at least 90% of surveyed genomes. The genes of this extended core comprise approximately 65% of individual *Aeromonas* genomes.

### Identification of T3SS related proteins in *Aeromonas* spp. genomes

Reference amino acid sequences of the T3SS apparatus and effector proteins were downloaded from the VFDB in April 2015. All *Aeromonas* spp. protein families were compared against the VFDB reference sequences using the HMMER suite [44]. HMMER profiles were generated for all identified homologous groups of *Aeromonas* proteins, with alignments performed using MAFFT [45]. Protein families without a positive VFDB hit (e-value > 1e-10) were dismissed from further investigation. Proteomes of *V*. *fisherii* ES114 [46] and *E*. *coli* K12 [47,48] were used as negative controls as they are known avirulent bacteria unlike to possess toxins in general and to not encode a T3SSs. We reasoned that by removing homologous groups matching equally well, or better, to the negative controls as to the VFDB sequences that the inclusion of distant T3SS homologs, such as F-ATPase or flagellar components, would be avoided. All protein sequences from groups representing positive hits against the VFDB were used as queries in BLAST searches against the two different data sets, the VFDB reference sequences and the negative control. A t-test was performed on bitscore distributions from homologous groups that had at least three positive hits against both the VFDB and the negative control to evaluate if the sequences were significantly more similar to those in the VFDB compared to the negative control (*p*≤0.05). In cases where the homologous group had less than three hits against one of the datasets, we assessed if the lowest alignment bitscore from the VFDB was at least 1.5-fold greater than that observed from the negative control. The Python scripts used to analyze both HMMER and BLAST outputs is available at github.com/lthiberiol/aeromonas_t3ss.

Gene families that were predicted to be related to the T3SS were manually curated and divided into separate clusters or merged into one as necessary. When two gene families contained corresponding domains but differed in the length of the ORF, their name was amended with “.1”. Families corresponding to structural components of T3SS-1 and T3SS-2 known to occur in *Aeromonas* [21] were identified. All putative effector families were manually identified from the set of 127 T3SS-related homologous groups. The correlations between effector occurrences (S1 Fig) were assessed using Pearson’s correlation tests and p-values corrected using Benjamini-Hochberg’s False Discovery Rate (FDR) correction (*q*≤0.05). The candidate effectors rarefaction curve was generated by assessing the average number of distinct candidate effectors present in 10,000 random combinations of *N* genomes, where *N* gradually increases from two to 105.

The final set of 23 putative T3SS effectors were submitted to additional assessment of their T3SS signal using web applications of both EffectiveT3 2.0.1 [33,49] and Bean 2.0 [50] with the default parameters.

### Reference phylogeny

Multiple sequence alignments were generated for all 5,693 single copy gene families using MAFFT, and quality control and edits were automatically performed using GUIDANCE [51]. Pairwise maximum likelihood distance matrices were calculated for gene families present in at least 10 genomes using Tree-Puzzle [52]. Pearson’s correlation tests between all gene family combinations were performed, and the significance of each correlation was assessed through Mantel tests and p-values that were corrected for multiple testing using Benjamini-Hochberg’s FDR correction. The correlation-weighted network (*q*≤0.05 and *rho*≥0.7) was submitted to the Markov Clustering Algorithm (MCL) with an inflation value of 5.5 [53,54]. The unpartitioned concatenation of the 1,678 gene families present in the largest cluster was submitted to RAxML [55] for phylogenetic reconstruction using the GTR+GAMMA substitution model, with support assessed using the aLRT SH-like method [56].

### Gene phylogenies and reconciliations

Nucleotide sequences from all putative effector gene families were aligned using MAFFT, and phylogenetic trees were generated using RAxML (GTR+GAMMA+I model). The obtained trees were refined using the TreeFixDTL tool [57] and reconciled to the species tree using RangerDTL [58]. S2 Fig shows cladograms of putative effector phylogenies after applying the TreeFixDTL tool and rooting the gene trees through RangerDTL.

### Addition of non-*Aeromonas* representatives

Amino acid sequences from the putative T3SS effectors were used to query against GenBank to identify non-*Aeromonas* homologs. The top non-*Aeromonas* hit was downloaded if the aligned region spanned through at least 60% of the query sequence and had an identity of at least 30%. Once a non-*Aeromonas* hit fulfilling the requirements was identified, it was downloaded together with all other non-*Aeromonas* hits with bitscores of at least 85% of the top hit.

### Strains and growth conditions

The bacterial and yeast strains and plasmids generated in this study are listed in S1 Table. The *E*. *coli* strain DH5α λ-*pir* was used to clone the plasmids and was cultured in LB broth or on agar solidified plates containing 100 μg/ml ampicillin as needed. The yeast strain *S*. *cerevisiae* BY4741 was cultured using yeast extract-peptone-dextrose (YPD) medium for non-selective growth[59], and selective growth was performed using synthetic defined medium lacking histidine (SDM-His) and containing either 2% glucose (SDM-His-Glu), or 2% galactose and 1% raffinose (SDM-His-Gal). Some strains were additionally evaluated on SDM-His-Gal medium supplemented with 7 mM caffeine or 0.5 M NaCl.

### Strain and plasmid construction

All primers used in the present study are listed in S4 Table. Putative T3SS effector genes were PCR amplified from *Aeromonas* genomic DNA using Phusion DNA polymerase (New England Biolabs; NEB) and appropriate primers. Primers were tailed to allow subsequent Gibson Assembly (NEB) cloning of amplicons at the *Eco*RI and *Xho*I sites of the shuttle vector pGREG533 (Euroscarf), a plasmid which allows for galactose inducible expression of cloned genes from the GAL1 promoter. Amplicons were purified using a Wizard SV Gel and PCR Clean up System kit (Promega) and *Eco*RI/*Xho*I digested pGREG533 was gel purified using a Qiaex II kit (Qiagen). The effector genes were cloned in-frame with the N-terminal 7× hemagglutinin tag (7-HA) in pGREG533. Constructs with inserts of the correct size were initially screened for by PCR and were subsequently sequenced and transformed into *S*. *cerevisiae* strain BY4741. Yeast were transformed by mixing 0.1 μg of plasmid, 0.1 mg of salmon sperm carrier DNA (Invitrogen) and 0.1 ml of yeast cells resuspended in sterile 1× TE/LiAC buffer (0.1 M Tris-HCl, 10 mM EDTA, and 0.1 M LiAc, pH 7.5). After adding 0.6 ml of sterile 1× TE/LiAc buffer containing 40% polyethylene glycol 4000, the cells were incubated at 30°C for 30 min with shaking (200 rpm). Next, the suspensions were mixed with 70 μl of dimethyl sulfoxide (DMSO), heat shocked at 42°C for ≥ 15 min, and then were centrifuged and resuspended in sterile water. Cell dilutions were plated on SDM-His-Glu plates and incubated for two days at 30°C to obtain transformants.

### Yeast growth inhibition assay

For each strain, 3 ml of SDM-His-Glu broth was inoculated with a single yeast colony and incubated at 30°C overnight with shaking. After centrifugation at 800 × *g* for 5 min, the supernatant was decanted and the cell pellet resuspended in sterile water. This process was repeated to remove residual medium, after which the cells were diluted to an OD_600_ of 1.0 and four 10-fold serial dilutions were made. A 10 μl aliquot of each dilution was spotted onto SDM-His-Glu plates or SDM-His-Gal. Yeast strains presenting partial (*aopP*, *pteH*, *pteJ* and *pteL*) or no growth inhibition phenotypes (*aopH*, *aopO*, *pteD*, *pteD*.*1*, *pteE* and *pteK*) on SDM-His-Gal plates were further assessed on SDM-His-Gal plates containing 7 mM caffeine or 0.5 M NaCl. The plates were incubated at 30°C for 2–3 days and were photographed with a Nikon D80 camera. Candidate effectors were cloned into pGREG533 in a galactose inducible manner from the GAL1 promoter. Plasmids expressing representatives of 21 out of the 23 previously identified or putative effector proteins were separately introduced into the yeast strain *S*. *cerevisiae* BY4741. 10-fold serial dilutions of cells were spotted onto agar-solidified plates that repressed (SDM-His-Glu) or promoted (SDM-His-Gal) the expression of the protein of interest. Representatives from two candidate effectors, *pteM* and *pteM*.1, were not experimentally evaluated because they were identified within incomplete ORFs.

### Western blot analysis

The expression of putative T3SS effectors in yeast strains presenting no growth inhibition phenotypes under any of the assayed conditions (*aopH*, *aopO*, *pteD*, *pteD*.*1*, *pteE* and *pteK*) was assessed by western blotting. The strains were inoculated from plates into SDM-His-Glu broth and grown overnight at 30°C with shaking (200 rpm). One milliliter of each culture was pelleted and resuspended in 300 μl of lysis buffer and added to a bead beating tube containing 300 mg of 0.5 mm zirconia/silica beads (cat# 11079105z, BioSpec Products) (50 mM Tris-HCL, 1% DMSO, 100 mM NaCl, 1 mM EDTA, and 1 mM PMSF, pH 8.0). The samples were homogenized 2× at 2000 rpm for 20 seconds and 4× at 4000 rpm for 10 seconds using a Qiagen Powerlyzer 24, with the samples placed on ice between each round of bead beating. The beads were pelleted by centrifuging the samples at 16,000 × *g* for 1 minute, after which supernatants were mixed with an equal volume of Laemmli buffer and were heated for 5 min at 100°C. The protein extracts were separated by SDS-PAGE on a 4–20% Mini Protean TGX gel (Bio-Rad) and then transferred to a PVDF membrane. The N-terminal 7-HA-tagged proteins were probed for using a mouse monoclonal anti-HA antibody followed by an HRP-conjugated goat anti-mouse IgG H&L HRP secondary antibody (ab49969 and ab205719, respectively; Abcam). The blots were developed using an ECL Plus Western Blotting Substrate kit (Pierce) and imaged using a FluorChem HD2 (ProteinSimple).

## Results and discussion

### Reference phylogeny

A phylogenomic study of 56 high-quality genomes from *Aeromonas* strains was published together with an MLSA tree of 16 housekeeping genes from the same genomes [40]. This study identified incongruities between gene, MLSA, and core genome trees [60], which is expected since different sets of genes were used to construct each tree. In our current study, we expanded on this set with an additional 49 *Aeromonas* genomes. To produce a reference phylogeny that reflects a significant share of *Aeromonas* genes and minimize conflicting evolutionary signals among genes, we constructed a phylogenomic tree using 1,678 genes with compatible evolutionary histories. Building phylogenies from concatenated genes is a widely used approach to reconstruct the representative history of a genome. However, the concatenation of genes with different trajectories can lead to unresolved trees and/or a phylogeny that represents neither the history of the organism nor that of the individual genes used in the concatenation [61–64]. One approach used to obtain better phylogenies is to group gene families with compatible evolutionary histories using tree distance metrics [65,66]. Therefore, to assess the similarity between the evolutionary histories of different genes, we performed pairwise Pearson’s correlation tests between Maximum Likelihood distance matrices of all gene families present in at least 10 genomes. Phylogenetic tree distance metrics that account only for branch lengths have been previously shown to perform as well as metrics accounting for both branch length and topology [66]. For comparison, we also measured the distances between gene families using the Fitch-Margoliash criterion [67], i.e., in calculating the sum of squares of the differences in distance between two matrices, the square of each difference was multiplied by the inverse of the distance, thereby increasing emphasis on the difference in distance between more similar sequences. The two approaches used to compare distance matrices showed a strong correlation (*r_s_* = −0.89). Significant correlation coefficients (*q*≤0.05) were submitted to a Markov Clustering (MCL) process [54], and a weighted network of connected gene families were constructed as discussed by van Dongen and Abreu-Goodger [68]. From the 168 gene family clusters obtained through MCL clustering, we considered the largest cluster, which contains 1,678 gene families, to be the primary phylogenetic signal among *Aeromonas* strains. This gene family cluster comprises more than twice the number of gene families present in the second largest cluster, which contains 738 gene families. By using the largest gene cluster that contained gene families with different functions and genomic locations but with compatible evolutionary histories, we ensured that our phylogeny is representative of the evolutionary history of the genomes.

**Fig 1:**
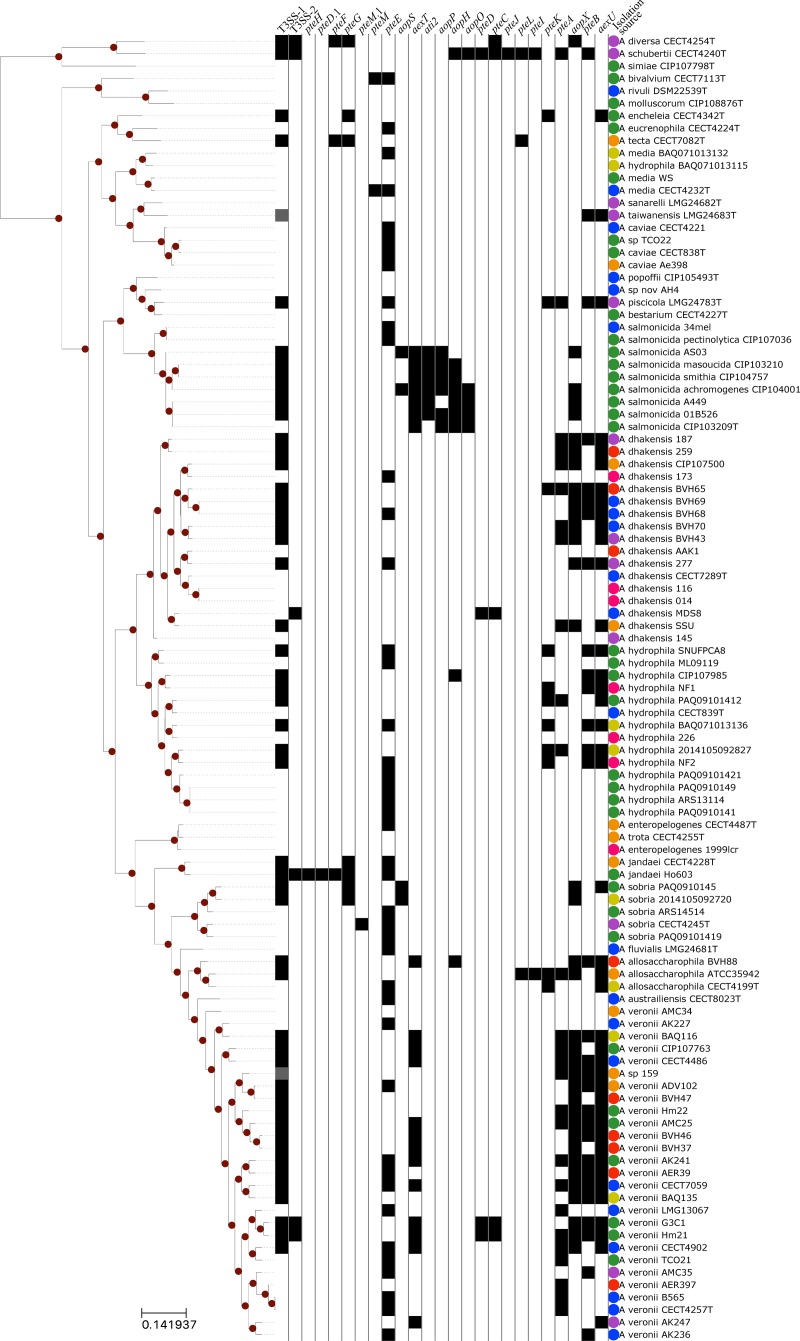
Phylogeny of 105 *Aeromonas* spp. genomes. Tree branches with sh-like aLRT support of at least 90% are highlighted with red circles. The binary heat map represents the presence/absence of T3SS apparatus and identified putative effectors in *Aeromonas* spp. isolates. The dark gray squares in the T3SS-1 column denote partial presence of the apparatus due to missing genes. The isolation source colors represent: green, veterinary; yellow, sick veterinary; blue, environment; orange, feces; red, blood; purple, wound; and pink, human.

On average, each *Aeromonas* genome has homologs from 1,637 out of the 1,678 families used in the reference phylogeny (*std* = 63.7), representing 39% of the average number of coding sequences in the *Aeromonas* species assayed. The resulting phylogenomic tree (Fig 1) is highly supported, with all but three branches displaying an aLRT SH-like support [56] greater than 90%. The concatenated gene tree presented in this study is largely consistent with the previously published *Aeromonas* spp. core-genome phylogeny [40], with the only differences being the deeper branching of *A*. *salmonicida* strains in relation to the common ancestor of *A*. *hydrophila* and *A*. *veronii*, as well as *A*. *allosaccharophila* being a sister group of *A*. *veronii*.

### *In silico* identification of putative T3SS effectors

For the identification of putative effectors, initially all coding sequences from sampled genomes were compared to each other to cluster homolog genes. Hidden Markov Model (HMM) profiles generated from each of the 25,518 identified *Aeromonas* spp. homolog gene families were queried against T3SS sequences from the VFDB, which yielded 633 positive hits (e-value ≤ 1e-10). Although some of these positive hits were clearly not T3SS related they displayed alignments with e-values as low as 6*e*^−155^ due to a common origin of other systems shared with T3SS components. Protein sequences from *Vibrio fisherii* ES114 [46] and *Escherichia coli* K12 [48] were used as negative controls for bona fide T3SS sequences, as both species are avirulent, not known to have a T3SS, and unlikely to encode any type of toxin. The combination of BLAST searches of sequences from the 633 initial candidates against both VFDB and the negative control genomes identified 127 gene families significantly more similar to T3SS sequences. Through subsequent manual curation, we assessed the domains present in each putative family and their genomic contexts (*e*.*g*., whether they are adjacent to chaperones) and classified 23 gene families as encoding likely effectors (Table 1). The nucleotide and amino acid sequences for each of these gene families are available in S1 Dataset. It is undoubtedly possible that T3SS effector proteins remained undetected using our method. There are two reasons for a potential failure to identify a real effector. (A) Our approach likely discriminates effectors that are not represented in the Virulence Factors DataBase. (B) More effectors likely will be discovered when additional *Aeromonas* genomes are added to the analysis. To estimate the latter, we calculated a rarefaction curve for the number of identified effectors against the number of genomes analyzed (S3 Fig). Although the saturation curve still trends upwards at its right end, it does so gradually. Given this slope, it was necessary to increase the number of sampled genomes from 52 to 105 to increase the average number distinct effectors from 20 to 23.

**Table 1:** Relevant characteristics of putative T3SS effector groups. Number of proteins describes the total number of protein-encoding genes identified across all assayed genomes for a given group.

Effector	# of proteins (# of T^$^)	Length in bp (max, median, min)^$ $^	Pfam domains ^$ $ $^	Domain descriptions ^$ $ $^	# of proteins with a type III secretion signal^$ $ $ $^	GOterms
**PteH**	1 (0–0)	1383	PF01734	Patatin-like phospholipase	1, 1	lipid metabolic process
**PteD.1**	1 (0–0)	1032	N/A	N/A	0, 1	N/A
**PteF**	3 (2–0)	1059, 969, 963	PF03543	Yersinia/Haemophilus virulence surface antigen	2, 3	cysteine-type endopeptidase activity, pathogenesis
**PteG**	7 (0–2.79)	2010, 1872, 1833	PF01734	Patatin-like phospholipase	5, 7	lipid metabolic process
**PteM.1**	1 (0–0)	1098	PF03496	ADP-ribosytransferase exoenzyme	0, 1	extracellular region, pathogenesis
**PteM**	2 (1–1)	678, 615, 552	PF03496	ADP-ribosytransferase exoenzyme	1, 2	extracellular region, pathogenesis
**PteE**	51 (0–31.9)	1443, 1128, 1083	PF13776; PF02661	Domain of unknown function (DUF4172); Fic/DOC family	4, 1	N/A
**AopS**	5 (0–1)	1155, 1155, 1149	PF02661	Fic/DOC family	0, 5	N/A
**AexT**	20 (5–5)	1429, 1353, 1287	PF03496; PF03545	ADP-ribosyltransferase exoenzyme; Yersinia virulence determinant (YopE)	9, 17	extracellular region, pathogenesis
**Ati2**	6 (0–0)	1488, 1488, 1242	PF03372	Endonuclease/Exonuclease/ phosphatase family	6, 5	N/A
**AopP**	6 (0–0)	897	PF03421	YopJ Serine/Threonine acetyltransferase	6, 5	N/A
**AopH**	9 (0–3)	1392, 1206, 1152	PF00102; PF09013	Protein-tyrosine phosphatase; YopH, N-terminal	1, 9	protein tyrosine phosphatase activity, protein dephosphorylation, protein tyrosine phosphatase activity, protein dephosphorylation, pathogenesis
**AopO**	5 (0–1)	2188, 2187, 2187	PF09632; PF00069	Rac1-binding domain; Protein kinase domain	5, 4	protein kinase activity, ATP binding, protein phosphorylation
**PteD**	4 (0–2)	1089, 1083, 1083	N/A	N/A	0, 4	N/A
**PteC**	5 (0–2)	1044, 1044, 999	N/A	N/A	5, 5	N/A
**PteJ**	1 (0–0)	708	PF03536	Salmonella virulence-associated 28kDa protein	1, 1	N/A
**PteL**	3 (2–2)	1452, 1452, 1446	N/A	N/A	3, 3	N/A
**PteI**	2 (1–1)	1134, 1132.5, 1131	PF03497	Anthrax toxin LF subunit	0, 1	GO:0005576, GO:0008294, pathogenesis
**PteK**	11 (0–4.86)	1422	PF09013	YopH, N-terminal	11, 11	GO:0004725, GO:0006470, pathogenesis
**PteA**	27 (0–13.5)	2112, 2103, 1944	N/A	N/A	20, 27	N/A
**AopX**	36 (14–14)	972, 951, 948	N/A	N/A	36, 35	N/A
**PteB**	30 (4–18)	951, 903, 492	N/A	N/A	15, 28	N/A
**AexU**	42 (15–15)	1545, 1537.5, 768	PF03545	Yersinia virulence determinant (YopE)	24, 41	N/A

$: Duplication, transfer, and loss events were estimated using Ranger-DTL [58] (see the Materials and Methods for additional details). No duplication was predicted within the putative effectors assayed. The number of transfers (T) with 100% confidence and the mean number of transfer events is given in parentheses, respectively.

$$: In instances where the max, median and min nucleotide length of genes was identical, a single value is given.

$$$: Domains present in an effector group were identified using the Pfam database, and the PFAM associated Go terms are given.

$$$$: Obtained using EffectiveT3 2.0.1 and Bean 2.0, respectively.

Eight of the 23 candidate effectors had been previously identified in *Aeromonas* species, including *aexT* [17,19,69], *aexU* [29], *aopP* [70,71], *aopH* and *aopO* [72], *ati2* [73], as well as *aopX* and *aopS*, which were initially identified in *A*. *salmonicida* as pseudogenes [74]. We also detected *aopN*, which was shown in *Bordetella bronchiseptica* to have a dual role in controlling the secretion of translocator proteins and suppressing host immunity but was not cytotoxic [75]. However, since our choice of screening method is designed to detect cytotoxicity, this protein was excluded from further analysis.

The remaining 15 likely *Aeromonas* T3SS effectors had not been studied in any detail and were designated as putative T3SS effectors, which included *pteA*, *pteB*, *pteC*, *pteD*, *pteD*.*1*, *pteE*, *pteF*, *pteG*, *pteH*, *pteI*, *pteJ*, *pteK*, *pteL*, *pteM*, and *pteM*.1. The *pteD*.1 and *pteM*.1 effectors were combined with the *pteD* and *pteM* families, respectively, despite being originally classified into distinct homolog groups since they share significant sequence similarity, as assessed through PRSS [76] using the PAM250 scoring matrix. The comparison between PteD.1 from *A*. *jandaei* Ho603 and PteD from A. sp. MDS8 sequences resulted in a Z-score of 65.7, and the comparison between PteM.1 from *A*. *sobria* CECT4245T and PteM from *A*. *media* CECT4232T resulted in a Z-score of 1003. Besides significant sequence similarity, homologs from both the *pteD* and *pteD*.*1* families are likely related to the same chaperone homolog group, whose members were automatically grouped within a single homolog protein cluster. Representatives of the fifteen newly described putative T3SS effector families and the 8 previously described effector families were submitted to InterProScan [77] for domain identification and to EffectiveT3 2.0.1 and Bean 2.0 for T3SS signal assessment (Table 1). One candidate effector group (*pteE*) presented an especially weak T3SS signal, as a very low percentage of *pteE* homologs (7%) were identified using EffectiveT3 or Bean. Twenty-one out of the 23 identified putative T3SS effectors were subsequently assessed for cytotoxic effects through expression in *S*. *cerevisiae* strain BY4741. Two candidate effectors, *pteM* and *pteM*.1, were not evaluated experimentally because they were incomplete ORFs.

### Screening of putative T3SS effector proteins in yeast

**Fig 2:**
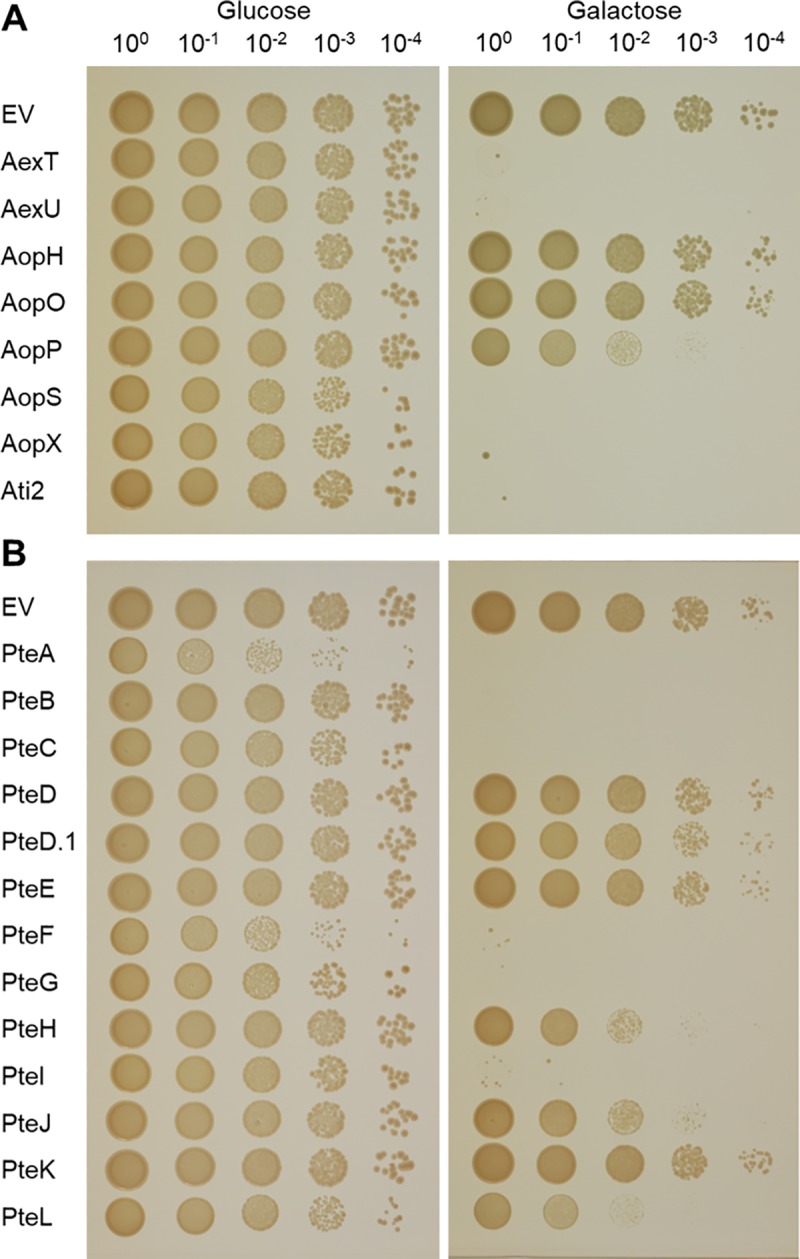
Yeast growth inhibition assay. Strains carrying putative T3SS effectors cloned into pGREG533 were cultured overnight in SDM-His-Glu, washed and 10-fold serially diluted. Aliquots from each dilution (10 μl) were spotted onto SDM-His-Glu and SDM-His-Gal plates. A strain containing pGREG533 was used as a negative control and showed no growth inhibition. SDM-His-Glu and SDM-His-Gal plates were incubated at 30°C for 2–3 days. T3SS effectors previously identified or biochemically characterized are presented in panel (A), whereas those identified in the present study are shown in panel (B).

To assess the cytotoxicity of putative T3SS effectors identified in *Aeromonas* spp. genomes, we expressed representative proteins from each putative group in the yeast strain *S*. *cerevisiae* BY4741 and monitored for growth inhibition (Table 1). Although eight of the identified *Aeromonas* effector families have previously been identified or studied, to the best of our knowledge, none have been assessed for causing cytotoxicity or growth inhibition in yeast. Expression of bacterial toxins in yeast is a common means of assessing the deleterious impact of these proteins on eukaryotic host cell processes [78–80]. The serial dilutions allow one to assess better how severe the cytotoxicity of an effector is. While all of the strains grew on medium containing glucose (Fig 2), those expressing *pteA* and *pteF* were somewhat inhibited for growth under non-inducing conditions, yielding colonies with reduced size relative to cells carrying the pGREG533 plasmid alone. Presumably, these effectors are so cytotoxic that even uninduced, basal protein expression is sufficient to inhibit growth. Interestingly, yeast cells transformed with *pteF* yielded colonies with different sizes, possibly due to the acquisition of suppressor mutations in the larger colonies that could assayed to identify potential targets of this protein.

Yeast strains expressing 15 of the 21 assayed proteins exhibited a growth phenotype when plated on SDM-His-Gal inducing medium (Fig 2). Strains expressing the proteins encoded by *aexT*, *aexU*, *aopS*, *aopX*, *ati2*, *pteA*, *pteB*, *pteC*, *pteF*, *pteG* and *pteI* showed little or no growth on the 10^0^ dilution (Fig 2), indicating that these effectors are very cytotoxic. In addition, strains expressing *aopP*, *pteH*, *pteJ*, and *pteL* exhibited reduced colony sizes with either no or little growth on the 10^−4^ dilution, suggesting that they are less cytotoxic. Strains expressing *aopH*, *aopO*, *pteD*, *pteD*.*1*, *pteE*, or *pteK* did not exhibit a growth inhibition phenotype compared to the control strain harboring pGREG533.

**Fig 3:**
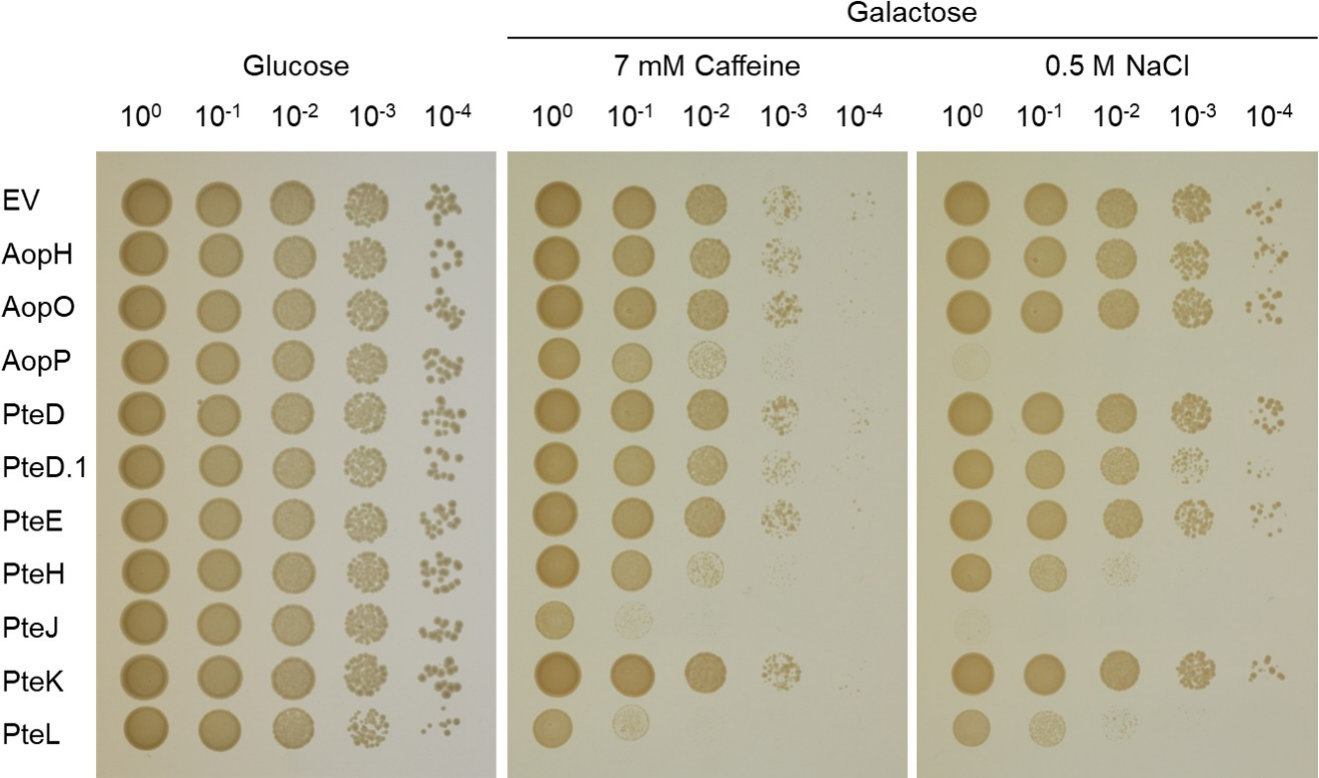
Yeast growth inhibition assay under stress conditions. Strains carrying putative T3SS effectors cloned into pGREG533 were grown overnight in SDM-His-Glu, washed and 10-fold serially diluted. Aliquots from each dilution (10 μl) were spotted onto SDM-His-Glu or on SDM-His-Gal plates containing either 0.5 M NaCl or 7 mM caffeine. The strain containing pGREG533 was used as a negative control and showed no growth inhibition. The plates were incubated at 30°C for 2–3 days.

If the cellular process targeted by a bacterial effector does not typically limit yeast growth, the presence of stressors (*e*.*g*., elevated salt or caffeine) can promote the inhibition phenotype to be observed [81]. The addition of NaCl to SDM-His-Gal medium inhibited the growth of strains expressing *aopP* and *pteJ* (slight growth on the 10^0^ dilution) compared to that observed on unsupplemented SDM-His-Gal medium (growth on the 10^4^ dilution) (Fig 3). On SDM-His-Gal plates containing caffeine, no further reduction in growth was observed for the *aopP*-expressing strain, whereas that of *pteJ*-expressing strain was observed on the 10^0^ and 10^−1^ dilutions (Fig 3). In addition, the strains expressing *pteH* and *pteL* produced small colonies at the 10^−3^ and 10^−2^ dilutions, respectively, when grown on SDM-His-Gal medium containing NaCl compared to that observed on SDM-His-Gal medium alone (growth on the 10^−3^ and 10^−2^ dilutions, respectively) (Fig 3). On plates containing caffeine, the strains expressing *pteH* and *pteL* grew on the 10^−3^ and 10^−1^ dilutions, respectively (Fig 3). For the constructs that did not produce a growth inhibition phenotype (*aopH*, *aopO*, *pteD*, *pteD*.*1*, *pteE*, and *pteK*), we assessed whether bacterial proteins of the expected fusion protein size were expressed by western blot and all of proteins were expressed at the predicted size except PteD for which no product was detected (S3 Fig).

Both AopH and PteK have similar domain structures (Table 1), and neither protein elicited a growth phenotype when expressed in yeast. The *Yersinia* homologs, YopH and YpkA, of these two *Aeromonas* proteins have been previously assayed in yeast for cytotoxicity [82]. Although YopH similarly did not cause a growth inhibition phenotype, YpkA strongly inhibit yeast growth. Several explanations could account for the lack of observed phenotypes in strains expressing AopH, AopO, PteD, PteD.1, PteE, and PteK, including: a lack of activity due to the adjoined 7-HA tag; the lack of a target protein for the effector in the yeast strain assayed; the effector does not produce a cytotoxic effect but interacts with host cells in another manner; the protein was not expressed at a high enough level; or the putative effector may not be a bacterial toxin.

These assays allowed the putative T3SS effectors identified in our bioinformatics analysis to be rapidly evaluated. Our findings showed that 15 out of the 21 tested proteins inhibited growth of *S*. *cerevisiae* BY4741, and that addition of NaCl, and to a lesser extent caffeine, to SDM-His-Gal plates increased the sensitivity of yeast cells to four of these likely effectors. Future assessments of the effectors with no observed phenotypes should focus on generating C-terminally tagged proteins, since the N-terminal tags generated in the current study could be the cause of aberrant activity or localization that may potentially mask a growth or cytotoxicity phenotype. Additionally, alternate yeast strains could be tested.

### Distribution and evolutionary history of putative effectors in *Aeromonas*

The scattered occurrence of the 23 predicted effectors described in this study throughout the *Aeromonas* phylogenomic tree is evidence of the impact of HGT during their evolution. Since only 15 out of the 23 putative effector homolog groups are present in at least four taxa, our HGT inferences using phylogenetic reconciliation are restricted to *aexT*, *aexU*, *aopX*, *aopO*, *aopP*, *ati2*, *aopH*, *aopS*, *pteA*, *pteB*, *pteC*, *pteD*, *pteE*, *pteG*, and *pteK*. In effectors present in less than four genomes, we only considered their presence/absence to infer gene transfer and loss events. The presence of a given putative effector among Aeromonads could be due to two possible scenarios: [1] vertical inheritance, or [2] HGT from a non-*Aeromonas* lineage. In a reconciliation scenario where no HGT is allowed among Aeromonads, 1,177 gene loss events and 105 gene duplication events are required to reconcile the histories of 15 putative effectors across *Aeromonas* phylogeny. In the reconciliation of the same 15 putative effectors allowing HGT and using default reconciliation penalties (loss = 1, duplication = 2, and transfer = 3), only 85 gene losses and 115 HGTs are required. Of the 115 predicted HGT events, 44 took place between terminal nodes of the tree. There is a significantly larger number of inferred HGTs between genomes from distinct species (30) than between members of the same species (14). Such small number of inferred within-species transfers may be due to a lack of resolution in the gene trees leading to inferred events with low confidence values. If a strong phylogenetic signal is absent from bipartitions present in the putative effector tree, our reconciliation approach assumes it to be equivalent to the genome phylogeny, since the gene tree bipartition does not strongly support the incongruence. Reconciliations also revealed a large variance of HGT events inferred among T3SS effector gene families, ranging from 0 to 32 horizontal transfers within *Aeromonas* spp. In addition, we assessed whether or not the predicted effectors co-occurred with a T3SS apparatus. Two distinct types of T3SS systems, here labelled as T3SS-1 and T3SS-2, have been described in Aeromonas [21]. Three effectors, PteE, PteM and PteM.1 were predominantly present in strains not carrying a T3SS apparatus (present in 69.77, 100 and 100% of strains, respectively), suggesting that they are not T3SS effectors. Interestingly, in addition to PteE, PteM, and PteM.1, eight candidate effectors were also detected in genomes not carrying a T3SS apparatus. A similar finding was reported among *Xanthomonas* genomes, were six out of 14 strains without T3SS structural genes possessed at least one effector [83]. While this could be due to the rapid acquisition of genes, including T3SS effectors, from bacteriophage [84], this result could also be due to the loss of a T3SS or the gain of effectors by other mechanisms.

To evaluate the gene exchange between *Aeromonas* spp. and other lineages, we recruited non-*Aeromonas* homologs of the 15 putative effectors present in four or more *Aeromonas* genomes. The inclusion of non-*Aeromonas* homologs revealed that the putative and previously described *Aeromonas* T3SS effectors are not frequently shared outside the genus boundaries. In all extended candidate effector trees, *Aeromonas* homologs grouped into clans [85], restricting homologs from other taxa to separate clades. This result suggests that each of the putative effectors has a single origin within *Aeromonas* strains, i.e., they entered the genus only once, either from its common ancestor or through a single HGT event. The disparity between within-genus and between-genera transfers is well described in the literature [20,86–88]. The high number of inferred gene transfers within *Aeromonas* genomes is evidence of the interchangeability of T3SS effectors within the genus, while the paucity of HGT events between genera may either reflect decreasing transfer rate with increasing evolutionary distance [87–89] or the high degree of specificity between effectors and the secretion system apparatus. We recognize the potential for undetected transfers with unsampled lineages not represented in public databases, although one would expect to observe at least some number of paraphyletic aeromonad clades if between-genera exchanges involving *Aeromonas* were common.

Five putative effector families exhibited more than ten HGT events within *Aeromonas* spp. according to phylogenetic reconciliations. The most exchanged effector is *pteE*, with 32 inferred transfers within the genus. We expect that the high number of HGT events involving *pteE* is unrelated to a possible role in the T3SS given its unrelated genomic occurrence unrelated with respect to the apparatus and a weak T3SS signal (Table 1). The reconciliation analysis inferred 18 HGT events during the evolution of *pteB* in *Aeromonas*, three HGT events more than identified among *aexU* homologs, despite the former being identified in 12 less genomes than the latter (Table 1). Two of the identified effectors, *aopP* and *ati2*, did not require horizontal transfers during its reconciliations with the genome phylogeny. Both effector families are exclusively present in *A*. *salmonicida* genomes, although *aopP* is absent from *A*. *salmonicida* A449, and *ati2* is absent from *A*. *salmonicida* CIP103209T, and their closest non-*Aeromonas* homologs are present in *Yersinia enterocolitica* and *Vibrio harveyi*, respectively. The genomes of *A*. *salmonicida* strains are very similar to each other, which is reflected in the short branch lengths present in the genome phylogeny (Fig 1). Thus, we would not expect a significant number of different HGT events among *A*. *salmonicida* strains. We hypothesize that an *A*. *salmonicida* common ancestor acquired these effector genes through horizontal transfer, likely from *Yersinia* spp. or *Vibrio* spp., which were then vertically inherited by most of the descendants.

The great variation in effector occurrence among *Aeromonas* strains, from zero to nine effectors per genome, may reflect the diversity of lifestyles observed in this genus. For example, *A*. *schubertii* possesses nine likely effectors in its genome, more than any other assessed strain, and it is also among the earliest branching aeromonads (Fig 1). We were unable to determine if this large number of predicted T3SS effectors is related to the niche *A*. *schubertii* occupies (cultured from a clinical forehead abscess), since no other sampled strain was isolated from a similar source. On the other side of the spectrum, 19 strains have no putative effector, and they are found across all clades of the *Aeromonas* phylogeny. Among putative effectors, some exhibit a wide distribution (*pteA* and *pteB*), whereas others have a very restricted distribution (*pteJ*, *pteI*, and *pteL*).

The majority of the putative effectors display significant co-occurrence with other putative effectors in *Aeromonas* genomes (*q*<0.05). One cluster of five putative effectors displays strong co-occurrence (*aexT*, *aopH*, *aopO*, *aopP* and *ati2*) (S1 Fig), and their occurrence is most prevalent in the *A*. *salmonicida* branch (Fig 1). Interestingly, one effector in this co-occurring cluster, *aexT*, is also present in *A*. *veronii* strains. Another co-occurring cluster comprises a distinct set of five putative effectors (*aexU*, *aopX*, *pteA*, *pteB* and *pteK*) (S1 Fig), and their occurrence is correlated with T3SSI-1 and mainly related to is primarily associated with three different clades of the phylogeny, including the *A*. *hydrophila*, *A*. *dhakensis*, and *A*. *veronii* clades. Genes encoding the T3SS-2 apparatus are present in only 6 *Aeromonas* genomes and exhibit diverse phylogenetic distribution, whereas *pteC* and *pteD* occur exclusively within genomes possessing T3SS-2. Due to the low frequency of other putative effectors with significant co-occurrences displayed in S1 Fig we cannot make reliable inferences based on their phylogenetic distribution. Despite the presence of co-occurring clusters, we are unable to identify links between presence/absence of putative effectors and isolation sources of their respective genomes. The resemblance of phylogenetic signal observed in co-occurrence of putative effectors is probably due to the higher within-species HGT frequency, as once the effector is acquired by a member of the species it is easily spread among closely related genomes [89,90]. Despite strong evidences described for 20 identified candidate T3SS effectors present in *Aeromonas* spp., the combination of weak T3SS secretion signal (Table 1) and the lack of co-occurrence with a T3SS apparatus (S1 Fig) for *pteE*, *pteM*, and *pteM*.1 suggest that members of these homolog groups are not T3SS effectors.

### Conclusions

In this study, we identified likely T3SS effectors present in the genomes of 105 *Aeromonas* strains and assessed their cytotoxicity in *S*. *cerevisiae*. The *in silico* identification of T3SS effector sequences has been considered to be a complex task given that their short sequences and shared homology with proteins associated with different cellular systems constitute a barrier to accurate analysis [49,91,92]. Our two-step comparisons against positive and negative data sets greatly reduced the number of false positives and resulted in the identification of 12 new likely *Aeromonas* effector families and eight that were previously described. The expression of these proteins in *S*. *cerevisiae* provided strong evidence for the cytotoxicity of most of the identified effectors.

The high frequency of horizontal transfer events of effectors within *Aeromonas* is reflected in their scattered distribution throughout the phylogenomic tree of the genus and reconciliations of each effector gene tree with the phylogenomic tree. Members of the *Aeromonas* genus are known for promiscuous gene exchange [20,93], as are genes associated with the T3SS [94,95]. A comparison of the number of DTL events necessary for gene tree reconciliations between scenarios where HGT was allowed or not provided strong evidence for the high exchange rate of putative T3SS effectors among aeromonads. The larger number of predicted inter- versus intra-species HGTs could be explained by vertical transmission being the dominant mode in any given species. In the context of our study we observed that sharing an isolation source had a smaller impact on effector distribution compared to the phylogenetic signal. This result could also be due to *Aeromonas* strains interacting with a wide range of eukaryotic hosts, with each strain requiring a different set of molecular tools. Again, this strong phylogenetic signal in T3SS effectors distribution reflects the importance of the vertical inheritance of effectors within closely related organisms.

Using a combination of bioinformatic and molecular approaches, we were able to identify nine new putative T3SS effectors that are toxic to yeast cells. Future studies should focus on further assaying the proteins identified in this study as potential T3SS effectors, including those that did not induce cytotoxicity in yeast or were not expressed, through the immunodetection of secreted proteins in defined medium and assays that examine their translocation in heterologous model systems. In addition, the role of individual effectors in different animal models, e.g., fish, mice, wax worms, and leeches would be useful to further characterize these proteins and perhaps identify specific niches that some are associated with. In addition, the bioinformatic approach that we described can be used to identify potential effectors in other genera and also be applied to other gene families.

## Supporting information

S1 Fig Co-occurrence correlation heat map. The heat map displays all significant co-occurrences (*α*≤0.05) between putative effectors in *Aeromonas* spp. isolates. The assessed correlation scale varies between 0.8 and -0.4. The four observed clusters are composed of: [1], *aexT*, *aopH*, *aopO aopP*, *aopS* and *ati2*; [2] *aexU aopX*, *pteA*, *pteB*, and *pteK*; [3] *pteF*, *pteH*, and *pteG*; and [4] *pteC*, *pteD*, *pteI*, *pteJ*, and *pteL*.
(PDF)

S2 Fig Phylogenies for the putative effectors. The depicted root corresponds to a most parsimonious DTL reconciliation. Numbers give percent bootstrap support calculated from 1000 samples using RAxML (GTR+GAMMA+I model).
(PDF)

S3 Fig Rarefaction curve of 23 candidate T3SS effectors among 105 *Aeromonas* spp. genomes. Scatter plot displays number of distinct candidate effectors present in each of the 10,000 random genome combinations, gradually increasing from 2 to 104. Black solid line shows the average number of distinct candidate effectors present in random genome combinations.
(PDF)

S4 Fig Western blot analysis of *S. cerevisiae* BY4741 lysates expressing the 7-HA epitope-tagged putative T3SS effectors *aopH*, *aopO*, *pteD*, *pteD.1 pteE* and *pteK*. The molecular weights of the of the major bands corresponded to those of the indicated effectors as determined by comparison with Kaleidoscope prestained standard (BioRad).
(PDF)

S1 Table Characteristics and accession numbers for the genomes first reported in this study. (PDF)

S2 Table List of the 105 *Aeromonas* strains used in this study. (PDF)

S3 Table Strains and plasmids used in this study. (PDF)

S4 Table Primers used in this study. (PDF)

S1 Dataset Nucleotide and amino acid sequences for each of the putative T3SS effector groups. (ZIP)
