# Supplementary material for: Identification and characterization of putative Aeromonas spp. T3SS effectors
Source: PLoS One. 2019 Jun 4;14(6):e0214035. doi: 10.1371/journal.pone.0214035 (PMC6548356; doi:10.1371/journal.pone.0214035)
Supplement: S1 Fig — The heat map displays all significant co-occurrences (α≤0.05) between putative effectors in Aeromonas spp. isolates. The assessed correlation scale varies between 0.8 and -0.4. The four observed clusters are composed of: [1], aexT, aopH, aopO aopP, aopS and ati2; [2] aexU aopX, pteA, pteB, and pteK; [3] pteF, pteH, and pteG; and [4] pteC, pteD, pteI, pteJ, and pteL. (PDF) [file pone.0214035.s001.pdf]

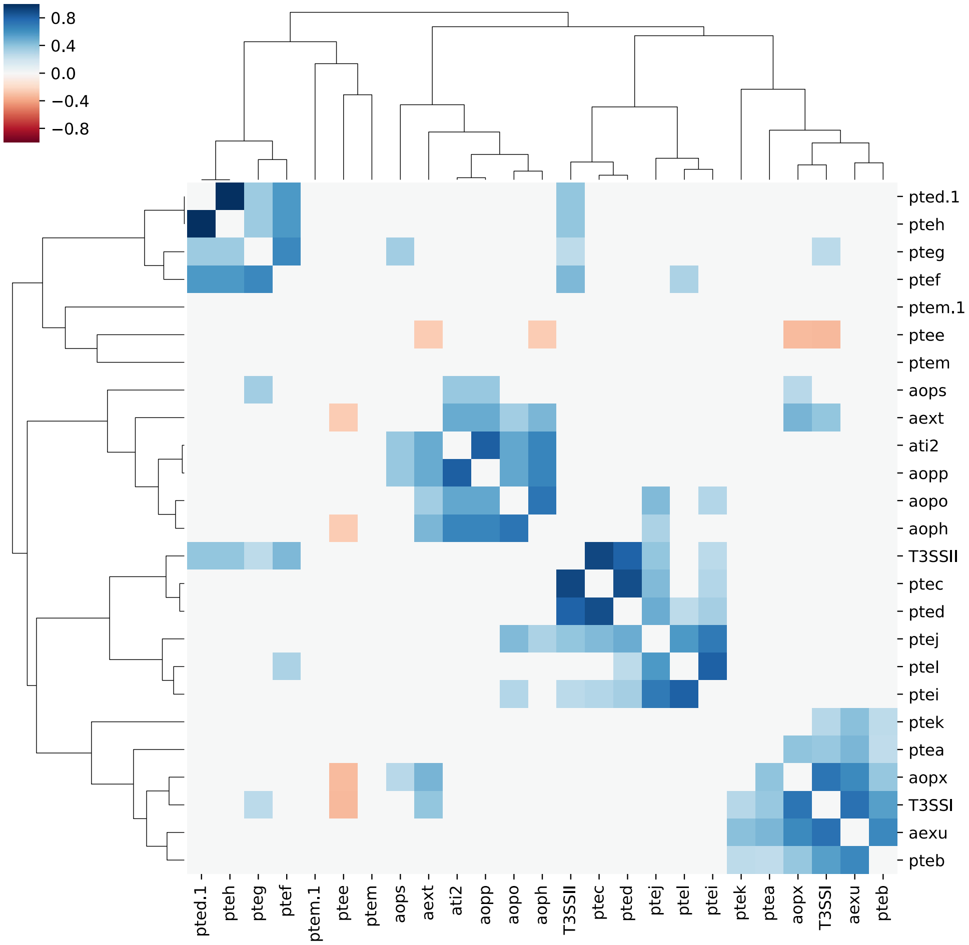

S1 Figure. Co-occurrence correlation heat map. The heat map displays all significant ($q \leq 0.05$) co-occurrences between putative effectors in *Aeromonas* spp. isolates. The assessed correlation scale varies between 0.8 and -0.4. The four observed clusters are composed by: [1] *aexT, ati2, aopP, aopH,* and *aopO*; [2] *pteK, aopX, pteA, pteB,* and *aexU*; [3] *pteH, pteG, pteD.1,* and *pteF*; and [4] *pteC, pteD, pteJ, pteL,* and *pteI*.
