# Supplementary material for: Identification and characterization of putative Aeromonas spp. T3SS effectors
Source: PLoS One. 2019 Jun 4;14(6):e0214035. doi: 10.1371/journal.pone.0214035 (PMC6548356; doi:10.1371/journal.pone.0214035)
Supplement: S2 Fig — The depicted root corresponds to a most parsimonious DTL reconciliation. Numbers give percent bootstrap support calculated from 1000 samples using RAxML (GTR+GAMMA+I model). (PDF) [file pone.0214035.s002.pdf]

AexT

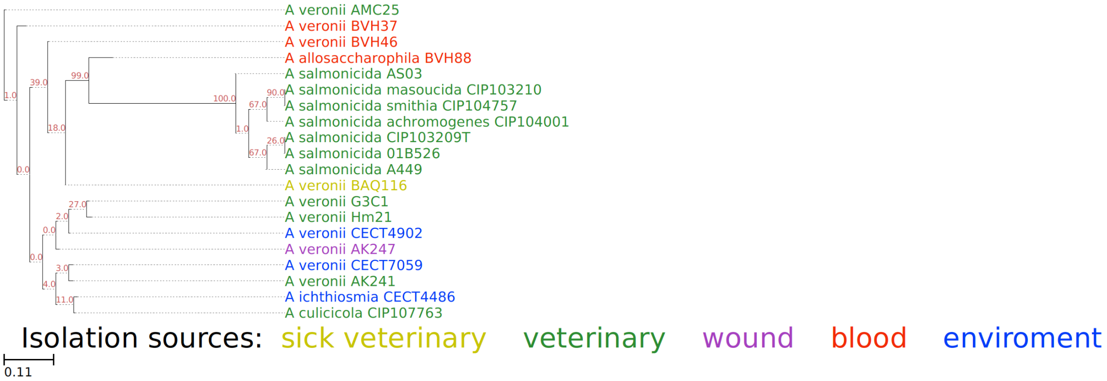

AexU

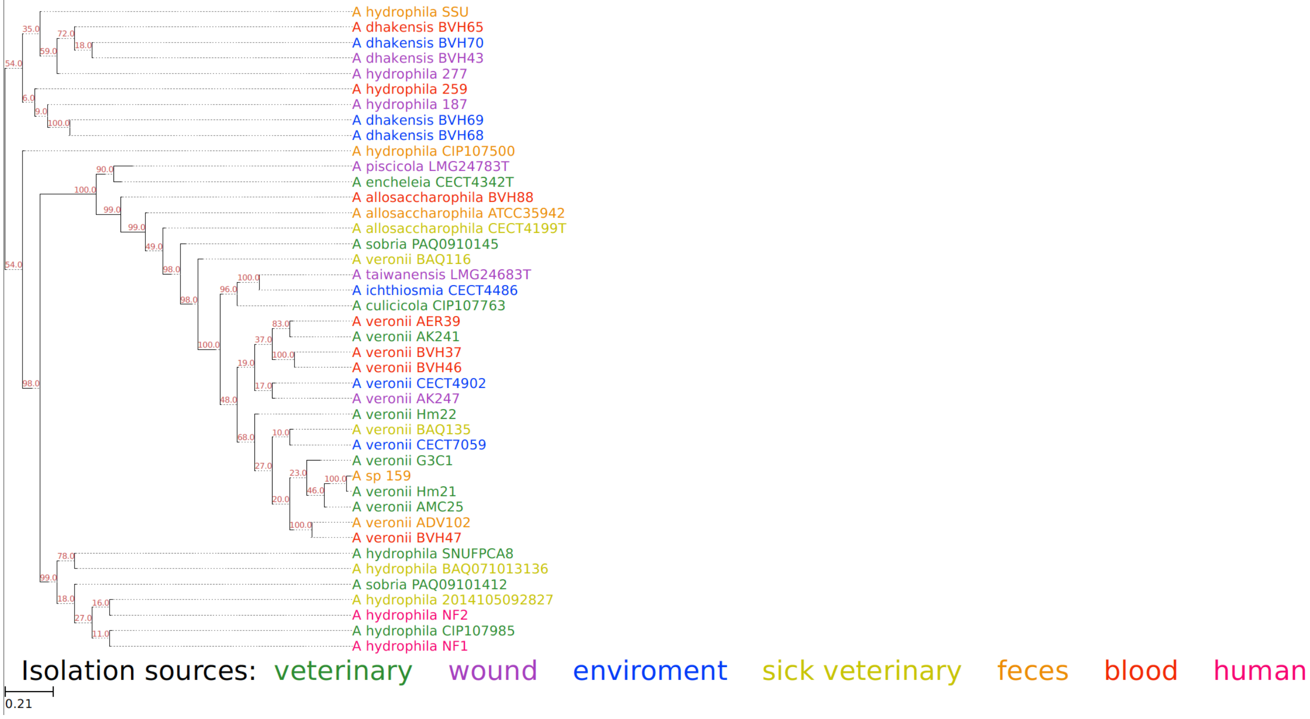

AopH

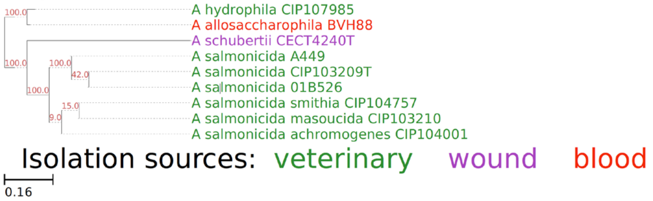

AopO

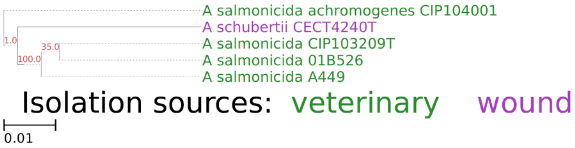

AopP

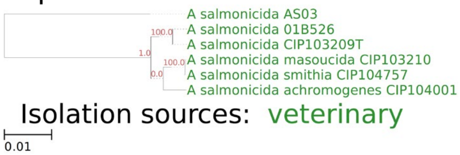

AopS

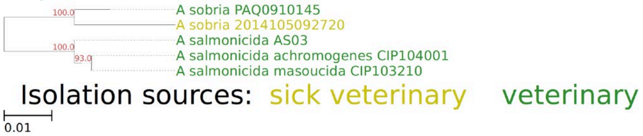

AopX

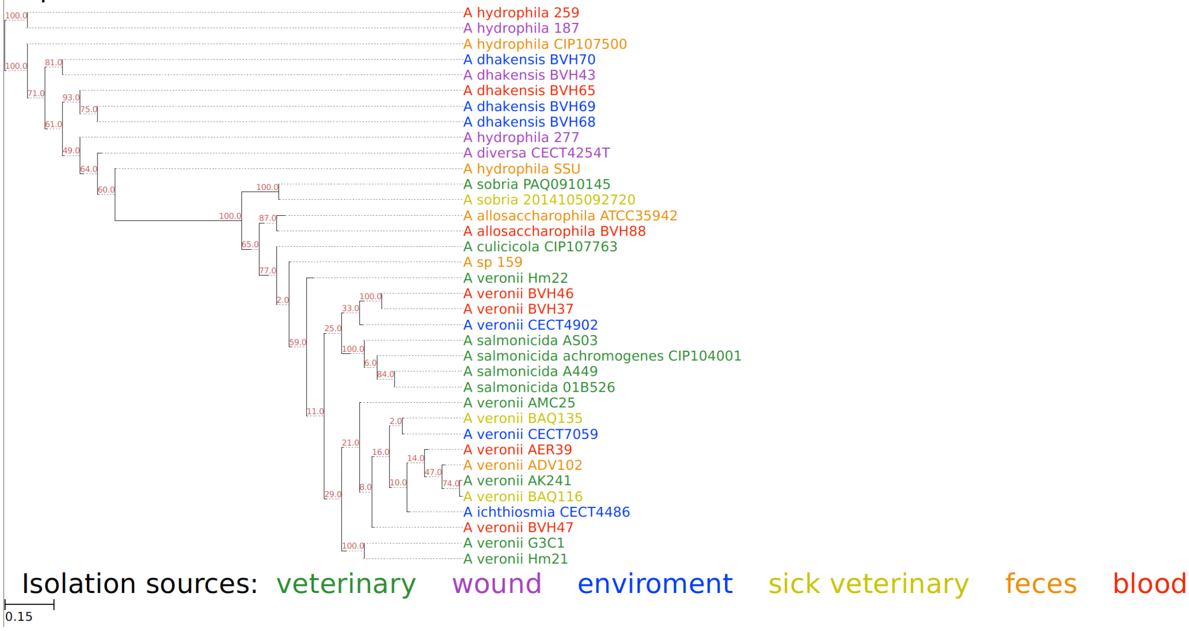

Ati2

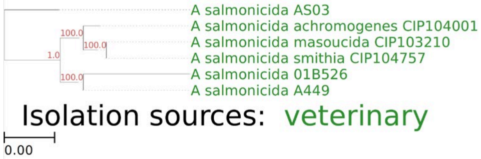

PteA

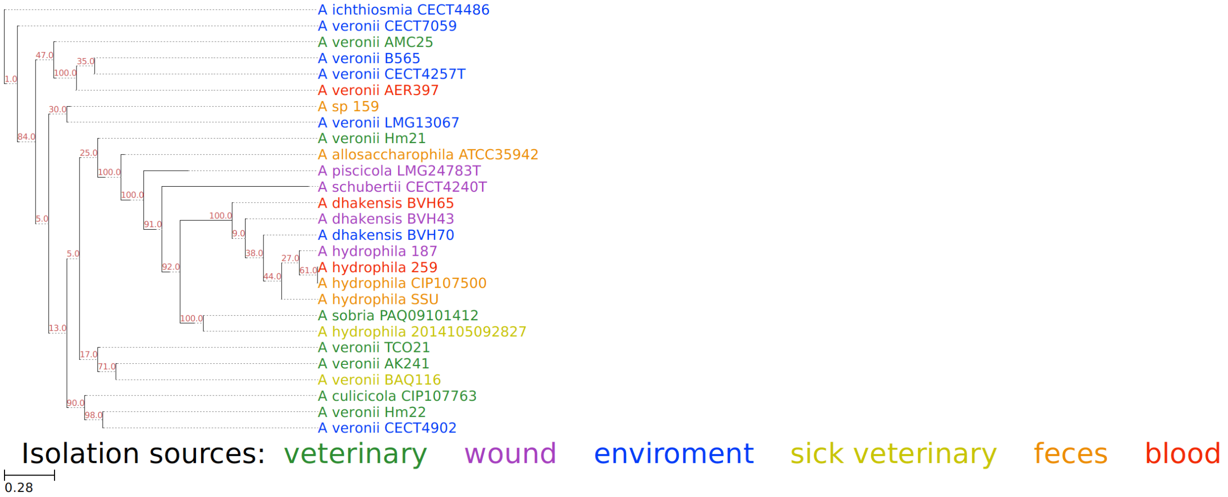

PteB

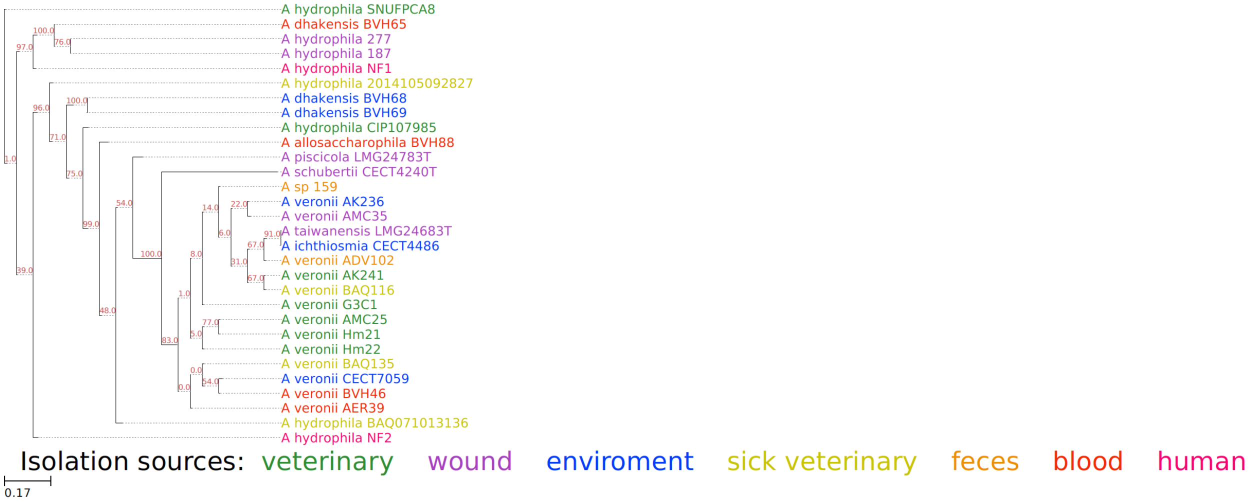

PteC

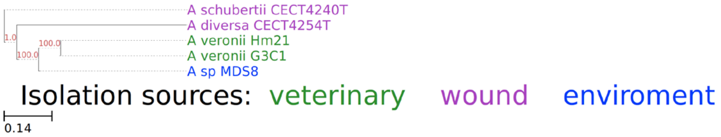

PteD

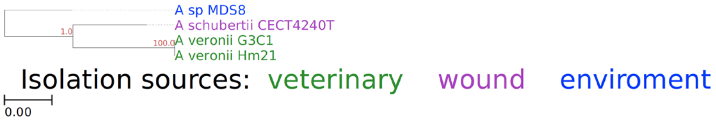

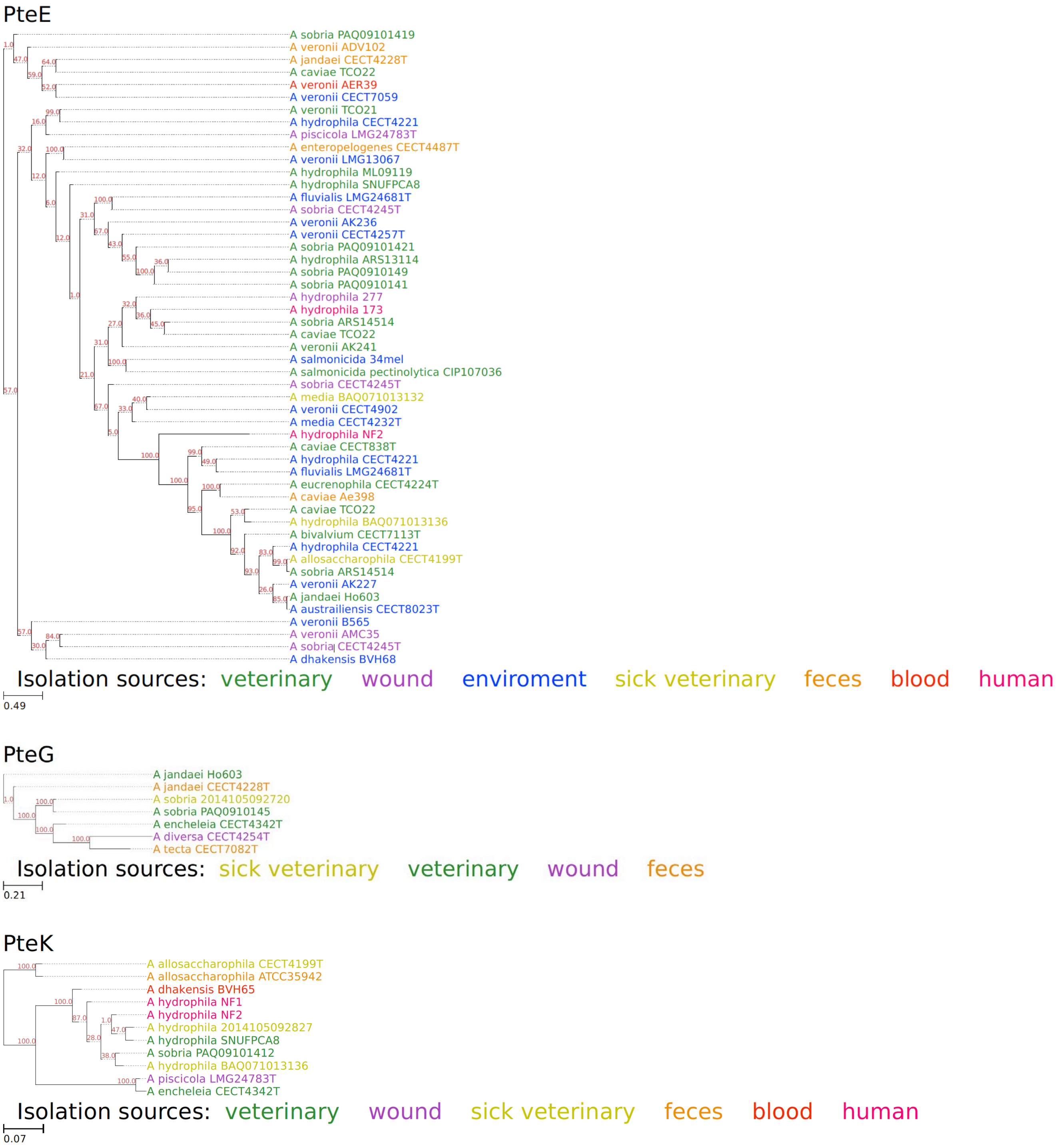

S2 Figure. Phylogenies for the putative effectors. The depicted root corresponds to a most parsimonious DTL reconciliation. Numbers give percent bootstrap support calculated from 1000 samples using RAxML (GTR+GAMMA+I model).
