# Supplementary material for: Identification and characterization of putative Aeromonas spp. T3SS effectors
Source: PLoS One. 2019 Jun 4;14(6):e0214035. doi: 10.1371/journal.pone.0214035 (PMC6548356; doi:10.1371/journal.pone.0214035)
Supplement: S3 Fig — Scatter plot displays number of distinct candidate effectors present in each of the 10,000 random genome combinations, gradually increasing from 2 to 104. Black solid line shows the average number of distinct candidate effectors present in random genome combinations. (PDF) [file pone.0214035.s003.pdf]

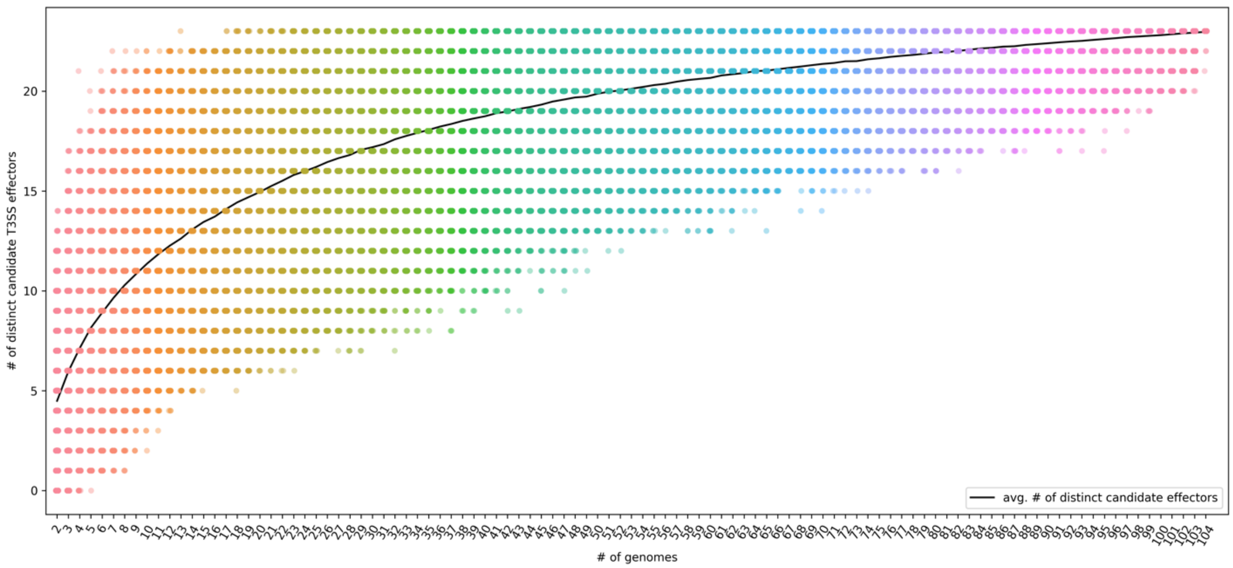

S3 Figure. Rarefaction curve of 23 candidate T3SS effectors among 105 *Aeromonas* spp. genomes. Scatter plot displays number of distinct candidate effectors present in each of the 10,000 random genome combination, gradually increasing from 2 to 104. Black solid line shows the average number of distinct candidate effectors present in random genome combinations.
