# Supplementary material for: Identification and characterization of putative Aeromonas spp. T3SS effectors
Source: PLoS One. 2019 Jun 4;14(6):e0214035. doi: 10.1371/journal.pone.0214035 (PMC6548356; doi:10.1371/journal.pone.0214035)
Supplement: S4 Fig — The molecular weights of the of the major bands corresponded to those of the indicated effectors as determined by comparison with Kaleidoscope prestained standard (BioRad). (PDF) [file pone.0214035.s004.pdf]

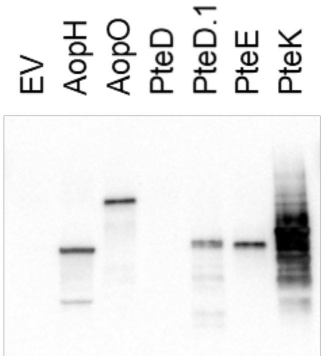

S4 Figure. Western blot analysis of *S. cerevisiae* BY4741 lysates expressing the 7-HA epitope-tagged putative T3SS effectors *aopH*, *aopO*, *pteD*, *pteD.1 pteE* and *pteK*. The molecular weights of the of the major bands corresponded to those of the indicated effectors as determined by comparison with Kaleidoscope prestained standard (BioRad).
