# Supplementary material for: Identification and characterization of putative Aeromonas spp. T3SS effectors
Source: PLoS One. 2019 Jun 4;14(6):e0214035. doi: 10.1371/journal.pone.0214035 (PMC6548356; doi:10.1371/journal.pone.0214035)
Supplement: S1 Table — (PDF) [file pone.0214035.s005.pdf]

S1 Table. Characteristics and accession numbers for the genomes first reported in this study.

Principle Strain Designation	Genome acc #	Biosample accession	Total Size (Mbp)	G+C %	No. Scaffolds	N50	Coverage
2014-10509-27-20	NKXJ00000000	SAMN07312741	4637050	57.8	200	45,353	*52*
2014-10509-28-27	NKXI00000000	SAMN07312742	4956224	61.1	107	136,771	97
ADV102	NKXH00000000	SAMN07312743	4526121	58.6	88	115,595	67
AK227	NKXG00000000	SAMN07312744	4406233	58.6	69	116,517	77
AK236	NKXF00000000	SAMN07312745	4414981	58.8	26	496,142	119
AK241	NKXE00000000	SAMN07312746	4599323	58.5	42	216,276	99
AK247	NKXD00000000	SAMN07312747	4551143	58.8	36	269,380	103
AMC25	NKXC00000000	SAMN07312748	4600367	58.8	50	203,652	64
ARS-131-14	NKXB00000000	SAMN07312749	4929129	60.9	70	135,668	78
ARS-145-14	NKXA00000000	SAMN07312750	4789633	56.6	100	112756	81
ATCC35942	NKWZ00000000	SAMN07312751	4539119	59.0	103	94,302	73
BAQ071013-115	NKWY00000000	SAMN07312752	4563682	62.3	89	94,956	93
BAQ071013-116	NKWX00000000	SAMN07312753	4575388	58.6	82	116,060	111
BAQ071013-132	NKWW00000000	SAMN07312754	4693239	61.3	167	54,818	42
BAQ071013-135		SAMN07312755	4622505	58.9	51	202,082	196
BAQ071013-136	NKWV00000000	SAMN07312756	4969114	60.9	84	126,127	129
BVH37	NKWU00000000	SAMN07312757	4467913	58.8	56	118,943	36
BVH43	NKWT00000000	SAMN07312758	4978043	61.4	57	179,181	83
BVH46	NKWS00000000	SAMN07312759	4519774	58.8	40	231,229	83
BVH47	NKWR00000000	SAMN07312760	4644947	58.9	109	100.022	80
BVH65		SAMN07312761	4787747	61.7	82	204,715	126
BVH68	NKWQ00000000	SAMN07312762	4860930	61.6	83	141,939	164
BVH69	NKWP00000000	SAMN07312763	4809752	61.6	62	179,245	126
BVH70	NKWO00000000	SAMN07312764	4720778	61.7	60	312,905	168
CECT4902	NKWN00000000	SAMN07312765	4642121	58.4	29	392,247	101
CECT7059	NKWM00000000	SAMN07312766	4805162	48.4	31	266,943	287
CIP103210	NKWL00000000	SAMN07312767	4537775	58.7	238	44,210	169
CIP104001	NKWK00000000	SAMN07312768	4620646	59.1	540	37,356	177
CIP104757	NKWJ00000000	SAMN07312769	4526273	58.7	234	44,203	92
CIP107036	NKWI00000000	SAMN07312770	4803087	58.5	225	59,159	151
Hm22	NKWH00000000	SAMN07312771	4928749	58.3	61	235,782	261
Ho603	NKWG00000000	SAMN07312772	4652860	58.5	183	47,469	40
PAQ091014-1	NKWF00000000	SAMN07312773	4937441	60.9	114	118,185	76
PAQ091014-12	NKWC00000000	SAMN07312776	4972821	61.4	95	119,732	55
PAQ091014-19	NKWB00000000	SAMN07312777	4679707	57.7	112	110,545	107
PAQ091014-21	NKWA00000000	SAMN07312778	4781067	61.3	68	138,524	99
PAQ091014-5	NKWE00000000	SAMN07312774	4661400	57.7	101	96778	150
PAQ091014-9	NKWD00000000	SAMN07312775	4927333	60.9	72	168,728	108
TCO21	NKVZ00000000	SAMN07312779	4464596	58.7	93	112,114	68
TCO22	NMSG00000000	SAMN07312780	4579713	61.2	179	57,828	51
