# Supplementary material for: Identification and characterization of putative Aeromonas spp. T3SS effectors
Source: PLoS One. 2019 Jun 4;14(6):e0214035. doi: 10.1371/journal.pone.0214035 (PMC6548356; doi:10.1371/journal.pone.0214035)
Supplement: S2 Table — (PDF) [file pone.0214035.s006.pdf]

Table S2. List of genomes used in this study. Coding genes were predicted using RAST platform.

Strain	# of CDS	Isolation source	Reference
A. salmonicida achromogenes CIP104001	4394	veterinary	This study
A. allosaccharophila ATCC35942	4079	feces	This study
A. allosaccharophila BVH88	4295	blood	Colston et al., 2014
A. allosaccharophila CECT4199T	4173	sick veterinary	Colston et al., 2014
A. dhakensis AAK1	4274	blood	Wu et al., 2012
A. dhakensis CECT7289T	4266	environment	Colston et al., 2014
A. austrailiensis CECT8023T	3733	environment	Colston et al., 2014
A. bestarium CECT4227T	4223	veterinary	Colston et al., 2014
A. bivalvium CECT7113T	4910	veterinary	Colston et al., 2014
A. caviae Ae398	4058	feces	Beatson et al., 2011
A. caviae CECT838T	4081	veterinary	Colston et al., 2014
A. veronii CIP107763	4012	veterinary	Colston et al., 2014
A. dhakensis BVH43	4554	wound	This study
A. dhakensis BVH65	4368	blood	This study
A. dhakensis BVH68	4447	environment	This study
A. dhakensis BVH69	4393	environment	This study
A. dhakensis BVH70	4308	environment	This study
A. diversa CECT4254T	3711	wound	Colston et al., 2014
A. encheleia CECT4342T	4076	veterinary	Colston et al., 2014
A. enteropelogenes 1999lcr	3780	human	Dallagassa et al, unpublished
A. enteropelogenes CECT4487T	4054	feces	Colston et al., 2014
A. eucrenophila CECT4224T	4113	veterinary	Colston et al., 2014
A. fluvialis LMG24681T	3620	environment	Colston et al., 2014
A. dhakensis 014	4245	human	Chan et al., 2011
A. dhakensis 116	4239	human	Chan et al., 2011
A. dhakensis 145	4301	wound	Chan and Chan unpublished
A. dhakensis 173	4346	human	Chan et al., 2011
A. dhakensis 187	4340	wound	Chan et al., 2011
A. hydrophila 2014105092827	4500	sick veterinary	This study
A. hydrophila 226	4601	human	Chan and Chan unpublished
A. dhakensis 259	4267	blood	Chan et al., 2011
A. dhakensis 277	4356	wound	Chan et al., 2011
A. sp nov AH4	4453	environment	Colston et al., 2014
A. hydrophila ARS13114	4461	veterinary	This study
A. hydrophila BAQ071013115	4128	sick veterinary	This study

A. hydrophila BAQ071013136	4504	sick veterinary	This study
A. caviae CECT4221	4207	environment	Colston et al., 2014
A. hydrophila CECT839T	4274	environment	Seshadri et al., 2006
A. dhakensis CIP107500	4284	feces	Colston et al., 2014
A. hydrophila CIP107985	4268	veterinary	Colston et al., 2014
A. hydrophila ML09119	4491	veterinary	Tekedar et al., 2013
A. hydrophila NF1	4305	human	Grim et al., 2104
A. hydrophila NF2	4310	human	Grim et al., 2104
A. hydrophila SNUFPCA8	4529	veterinary	Han, Kim, Choresca, et al., 2013a
A. dhakensis SSU	4482	feces	The Broad Institute
A. veronii CECT4486	3997	environment	Colston et al., 2014
A. jandaei CECT4228T	4065	feces	Colston et al., 2014
A. jandaei Ho603	4187	veterinary	This study
A. media BAQ071013132	4210	sick veterinary	This study
A. media CECT4232T	4075	environment	Colston et al., 2014
A. media WS	3882	veterinary	Chai et al., 2012
A. molluscorum CIP108876T	3932	veterinary	Spataro et al., 2013
A. piscicola LMG24783T	4713	wound	Colston et al., 2014
A. popoffii CIP105493T	4331	environment	Colston et al., 2014
A. rivuli DSM22539T	4149	environment	Colston et al., 2014
A. salmonicida 01B526	4423	veterinary	Charette et al., 2012
A. salmonicida 34mel	4212	environment	Pavan et al., 2013
A. salmonicida A449	4613	veterinary	Reith et al., 2008
A. salmonicida AS03	4119	veterinary	Han, et al., 2013b
A. salmonicida CIP103209T	4442	veterinary	Colston et al., 2014
A. salmonicida pectinolytica CIP107036	4301	veterinary	This study
A. salmonicida masoucida CIP103210	4357	veterinary	This study
A. dhakensis MDS8	4328	environment	RayChaudhuri et al., 2013
A. sp 159	3075	feces	Chan et al., 2012
A. salmonicida smithia CIP104757	4252	veterinary	This study
A. sanarelli LMG24682T	3828	wound	Colston et al., 2014
A. schubertii CECT4240T	3808	wound	Colston et al., 2014
A. simiae CIP107798T	3654	veterinary	Colston et al., 2014
A. sobria 2014105092720	4194	sick veterinary	This study
A. sobria ARS14514	4328	veterinary	This study
A. sobria CECT4245T	4160	wound	Colston et al., 2014
A. hydrophila PAQ0910141	4468	veterinary	This study
A. hydrophila PAQ09101412	4558	veterinary	This study
A. sobria PAQ09101419	4207	veterinary	This study

A. hydrophila PAQ09101421	4286	veterinary	This study
A. sobria PAQ0910145	4256	veterinary	This study
A. hydrophila PAQ0910149	4456	veterinary	This study
A. tecta CECT7082T	4278	feces	Colston et al., 2014
A. caviae TCO22	4210	veterinary	This study
A. taiwanensis LMG24683T	4467	wound	Colston et al., 2014
A. trota CECT4255T	3917	feces	Colston et al., 2014
A. veronii ADV102	4111	feces	This study
A. veronii AER39	3947	blood	The Broad Institute
A. veronii AER397	3979	blood	The Broad Institute
A. veronii AK227	3983	environment	This study
A. veronii AK236	3942	environment	This study
A. veronii AK241	4133	veterinary	This study
A. veronii AK247	4105	wound	This study
A. veronii AMC25	4165	veterinary	This study
A. veronii AMC34	4090	feces	The Broad Institute
A. veronii AMC35	4019	wound	The Broad Institute
A. veronii B565	4054	environment	Li et al., 2011
A. veronii BAQ116	4098	sick veterinary	This study
A. veronii BAQ135	4174	sick veterinary	This study
A. veronii BVH37	4031	blood	This study
A. veronii BVH46	4078	blood	This study
A. veronii BVH47	4244	blood	This study
A. veronii CECT4257T	4070	environment	Colston et al., 2014
A. veronii CECT4902	4165	environment	This study
A. veronii CECT7059	4288	environment	This study
A. veronii G3C1	4328	veterinary	Beka et al, 2018
A. veronii Hm21	4334	veterinary	Bomar et al., 2013
A. veronii Hm22	4443	veterinary	This study
A. veronii LMG13067	4171	environment	Colston et al., 2014
A. veronii TCO21	4055	veterinary	This study

Beatson SA, das Graças de Luna M, Bachmann NL, Alikhan N-F, Hanks KR, Sullivan MJ, et al. Genome sequence of the emerging pathogen Aeromonas caviae. J Bacteriol. 2011;193: 1286–7.

Beka L, Fullmer MS, Colston SM, Nelson MC, Talagrand-Reboul E, Walker P, et al. Low-Level Antimicrobials in the Medicinal Leech Select for Resistant Pathogens That Spread to Patients. MBio. 2018;9.

Bomar L, Stephens WZ, Nelson MC, Velle K, Guillemin K, Graf J. Draft Genome Sequence of Aeromonas veronii Hm21, a Symbiotic Isolate from the Medicinal Leech Digestive Tract. Genome Announc. 2013;1: e00800-13-e00800-13

Chai B, Wang H, Chen X. Draft genome sequence of high-melanin-yielding Aeromonas media strain WS. J Bacteriol. 2012;194: 6693–4.

Chan K-G, Puthucheary SD, Chan X-Y, Yin W-F, Wong C-S, Too W-SS, et al. Quorum sensing in Aeromonas species isolated from patients in Malaysia. Curr Microbiol. 2011;62: 167–72.

Chan K-G, Puthucheary SD, Chan X-Y, Yin W-F, Wong C-S, Too W-SS, et al. Quorum sensing in Aeromonas species isolated from patients in Malaysia. Curr Microbiol. 2011;62: 167–72.

Chan K-G, Puthucheary SD, Chan X-Y, Yin W-F, Wong C-S, Too W-SS, et al. Quorum sensing in Aeromonas species isolated from patients in Malaysia. Curr Microbiol. 2011;62: 167–72.

Colston SM, Fullmer MS, Beka L, Lamy B, Gogarten JP, Graf J. Bioinformatic genome comparisons for taxonomic and phylogenetic assignments using Aeromonas as a test case. MBio. 2014;5: e02136.

Grim CJ, Kozlova E V, Ponnusamy D, Fitts EC, Sha J, Kirtley ML, et al. Functional genomic characterization of virulence factors from necrotizing fasciitis-causing strains of Aeromonas hydrophila. Appl Environ Microbiol. American Society for Microbiology; 2014;80: 4162–83.

Chan K-G, Puthucheary SD, Chan X-Y, Yin W-F, Wong C-S, Too W-SS, et al. Quorum sensing in Aeromonas species isolated from patients in Malaysia. Curr Microbiol. 2011;62: 167–72.

Chan K-G, Puthucheary SD, Chan X-Y, Yin W-F, Wong C-S, Too W-SS, et al. Quorum sensing in Aeromonas species isolated from patients in Malaysia. Curr Microbiol. 2011;62: 167–72.

Chan K-G, Puthucheary SD, Chan X-Y, Yin W-F, Wong C-S, Too W-SS, et al. Quorum sensing in Aeromonas species isolated from patients in Malaysia. Curr Microbiol. 2011;62: 167–72.

Chan K-G, Puthucheary SD, Chan X-Y, Yin W-F, Wong C-S, Too W-SS, et al. Quorum sensing in Aeromonas species isolated from patients in Malaysia. Curr Microbiol. 2011;62: 167–72.

Chan K-G, Puthucheary SD, Chan X-Y, Yin W-F, Wong C-S, Too W-SS, et al. Quorum sensing in Aeromonas species isolated from patients in Malaysia. Curr Microbiol. 2011;62: 167–72.

Reith ME, Singh RK, Curtis B, Boyd JM, Bouevitch A, Kimball J, et al. The genome of Aeromonas salmonicida subsp. salmonicida A449: insights into the evolution of a fish pathogen. BMC Genomics. 2008;9: 427.

Seshadri R, Joseph SW, Chopra AK, Sha J, Shaw J, Graf J, et al. Genome sequence of Aeromonas hydrophila ATCC 7966T: jack of all trades. J Bacteriol. 2006;188: 8272–82.

Spataro N, Farfán M, Albarral V, Sanglas A, Lorén JG, Fusté MC, et al. Draft Genome Sequence of Aeromonas molluscorum Strain 848TT, Isolated from Bivalve Molluscs. Genome Announc. 2013;1.

Tekedar HC, Waldbieser GC, Karsi A, Liles MR, Griffin MJ, Vamenta S, et al. Complete Genome Sequence of a Channel Catfish Epidemic Isolate, Aeromonas hydrophila Strain ML09-119. Genome Announc. 2013;1

Wu C-J, Wang H-C, Chen C-S, Shu H-Y, Kao A-W, Chen P-L, et al. Genome sequence of a novel human pathogen, Aeromonas aquariorum. J Bacteriol. 2012;194: 4114–5.
