# Supplementary material for: Identification and characterization of putative Aeromonas spp. T3SS effectors
Source: PLoS One. 2019 Jun 4;14(6):e0214035. doi: 10.1371/journal.pone.0214035 (PMC6548356; doi:10.1371/journal.pone.0214035)
Supplement: S3 Table — (PDF) [file pone.0214035.s007.pdf]

Table S3. Strains and plasmids used in this study.

Strain	Description	Reference or Source
Escherichia coli DH5α λ-*pir*	Cloning strain used to maintain plasmids	(Boccazzi et al., 2000)
Saccharomyces cerevisiae BY4741	MATα *his3Δ1 leu2Δ0 met15Δ0 ura3Δ0*	(Brachmann et al., 1998)
**Plasmid**		
pGREG533	AmpR, CEN plasmid with HIS3 marker, GAL1 expression of N-terminal 7× HA fusion protein	(Jansen et al., 2005)
pGREG533-*aexT*	Amp^R, HIS3; gene from *Aeromonas veronii* Hm21	This study
pGREG533-*aexU*	Amp^R, HIS3; gene from *Aeromonas veronii* Hm21	This study
pGREG533-*aopX*	Amp^R, HIS3; gene from *Aeromonas veronii* Hm21	This study
pGREG533-*aopO*	Amp^R, HIS3; gene from *Aeromonas salmonicida* subsp. *achromogenes* CIP104001	This study
pGREG533-*aopP*	Amp^R, HIS3; gene from *Aeromonas salmonicida* subsp. *achromogenes* CIP104001	This study
pGREG533-*ati2*	Amp^R, HIS3; gene from *Aeromonas salmonicida* subsp. *achromogenes* CIP104001	This study
pGREG533-*aopH*	Amp^R, HIS3; gene from *Aeromonas allosaccharophila* BVH88	This study
pGREG533-*aopS*	Amp^R, HIS3; gene from *Aeromonas sobria* PAQ091014-5	This study
pGREG533-*pteA*	Amp^R, HIS3; gene from *Aeromonas veronii* Hm21	This study
pGREG533-*pteB*	Amp^R, HIS3; gene from *Aeromonas veronii* Hm21	This study
pGREG533-*pteC*	Amp^R, HIS3; gene from *Aeromonas veronii* Hm21	This study
pGREG533-*pteD*	Amp^R, HIS3; gene from *Aeromonas veronii* Hm21	This study
pGREG533-*pteD.1*	Amp^R, HIS3; gene from *Aeromonas jandaei* Ho603	This study
pGREG533-*pteE*	Amp^R, HIS3; gene from *Aeromonas jandaei* Ho603	This study
pGREG533-*pteF*	Amp^R, HIS3; gene from *Aeromonas jandaei* Ho603	This study
pGREG533-*pteG*	Amp^R, HIS3; gene from *Aeromonas jandaei* Ho603	This study
pGREG533-*pteH*	Amp^R, HIS3; gene from *Aeromonas jandaei* Ho603	This study
pGREG533-*pteI*	Amp^R, HIS3; gene from *Aeromonas schubertii* CECT4240T	This study
pGREG533-*pteJ*	Amp^R, HIS3; gene from *Aeromonas schubertii* CECT4240T	This study
pGREG533-*pteK*	Amp^R, HIS3; gene from *Aeromonas allosaccharophila* ATCC35942	This study
pGREG533-*pteL*	Amp^R, HIS3; gene from *Aeromonas allosaccharophila* ATCC35942	This study

Boccazzi P, Zhang JK, Metcalf WW. Generation of dominant selectable markers for resistance to pseudomonic acid by cloning and mutagenesis of the ileS gene from the archaeon Methanosarcina barkeri fusaro. J Bacteriol. 2000;182: 2611–8. doi:10.1128/jb.182.9.2611-2618.2000

Brachmann CB, Davies A, Cost GJ, Caputo E, Li J, Hieter P, et al. Designer deletion strains derived from Saccharomyces cerevisiae S288C: a useful set of strains and plasmids for PCR-mediated gene disruption and other applications. Yeast. 1998;14: 115–32. doi:10.1002/(SICI)1097-0061(19980130)14:2<115::AID-YEA204>3.0.CO;2-2

Jansen G, Wu C, Schade B, Thomas DY, Whiteway M. Drag&Drop cloning in yeast. Gene. 2005;344: 43–51. doi:10.1016/j.gene.2004.10.016
