# Supplementary material for: Identification and characterization of putative Aeromonas spp. T3SS effectors
Source: PLoS One. 2019 Jun 4;14(6):e0214035. doi: 10.1371/journal.pone.0214035 (PMC6548356; doi:10.1371/journal.pone.0214035)
Supplement: S4 Table — (PDF) [file pone.0214035.s008.pdf]

Table S4. Primers used in this study.

Name	Sequence (5’-3’)	Use
aexT_pGREG533_GA_F	CGGACTATGCAGGAGGGAATTCGATGCAGATTCAAGCAAACAC	Cloning
aexT_pGREG533_GA_R	CGTGACATAACTAATTACATGACTCGAGGTACTATCTGGTCACGCCAG	Cloning
aexU_pGREG533_GA_F	CGGACTATGCAGGAGGGAATTCGATGCAGATTCAAGCAAACACCGTC	Cloning
aexU_pGREG533_GA_R	CGTGACATAACTAATTACATGACTCGAGTTACAGATAGCTCTCATCGACGCCG	Cloning
aopX_pGREG533_GA_F	CGGACTATGCAGGAGGGAATTCGATGGCCTTACCAGCAATCG	Cloning
aopX_pGREG533_GA_R	CGTGACATAACTAATTACATGACTCGAGTTAATCCTCCCGATGGGTG	Cloning
aopO_pGREG533_GA_F	CGGACTATGCAGGAGGGAATTCGATGAAGATCATCGGAACCAC	Cloning
aopO_pGREG533_GA_R	CGTGACATAACTAATTACATGACTCGAGTTAGCGGATCCACTCCTG	Cloning
aopP_pGREG533_GA_F	CGGACTATGCAGGAGGGAATTCGATGAATATACCCCCCATCC	Cloning
aopP_pGREG533_GA_R	CGTGACATAACTAATTACATGACTCGAGTCAAACGGATTTTCCATCATC	Cloning
ati2_pGREG533_GA_F	CGGACTATGCAGGAGGGAATTCGATGTCTACAATTCAAATTAATAGCC	Cloning
ati2_pGREG533_GA_R	CGTGACATAACTAATTACATGACTCGAGTTAAAGATTGGCAACGAACTG	Cloning
aopH_pGREG533_GA_F	CGGACTATGCAGGAGGGAATTCGATGACCTTAGCGATTCACTCTC	Cloning
aopH_pGREG533_GA_R	CGTGACATAACTAATTACATGACTCGAGTTATGCCCGCAGTAATGGGC	Cloning
aopS_pGREG533_GA_F	CGGACTATGCAGGAGGGAATTCGATGATCGAGTTTAAAAGTGTGGCC	Cloning
aopS_pGREG533_GA_R	CGTGACATAACTAATTACATGACTCGAGTTATTTGATCCCGTGCAGTG	Cloning
pteA_pGREG533_GA_F	CGGACTATGCAGGAGGGAATTCGATGAAAATTACCTCCCCG	Cloning
pteA_pGREG533_GA_R	CGTGACATAACTAATTACATGACTCGAGCCTCGGGATGACCTAGAC	Cloning
pteB_pGREG533_GA_F	CGGACTATGCAGGAGGGAATTCGATGGCTATTCATGGAGCTACC	Cloning
pteB_pGREG533_GA_R	CGTGACATAACTAATTACATGACTCGAGATGGCAAGGGTTACACCTTG	Cloning
pteC_pGREG533_GA_F	CGGACTATGCAGGAGGGAATTCGATGGAACACATTAGCCGTTC	Cloning
pteC_pGREG533_GA_R	CGTGACATAACTAATTACATGACTCGAGTCAGGCAAAGCTAAAACTGTG	Cloning
pteD_pGREG533_GA_F	CGGACTATGCAGGAGGGAATTCGATGGAGCTTTCTATCAAAGTGAAC	Cloning
pteD_pGREG533_GA_R	CGTGACATAACTAATTACATGACTCGAGTTATTTCGTCACTTTGAAACGG	Cloning
pteD.1_pGREG533_GA_F	CGGACTATGCAGGAGGGAATTCGATGGAAACAAATTTCAAATGTTTTAAATCG	Cloning
pteD.1_pGREG533_GA_F	CGTGACATAACTAATTACATGACTCGAGTCAACTGTTCTGAGGCATCATCG	Cloning
pteE_pGREG533_GA_F	CGGACTATGCAGGAGGGAATTCGATGAGGGCTATTATCGGCTC	Cloning

pteE_pGREG533_GA_R	CGTGACATAACTAATTACATGACTCGAGTCAGTGATTTGTCAATTTGAGC	Cloning
pteF_pGREG533_GA_F	CGGACTATGCAGGAGGGAATTCGATGATGATGACAATCACCTCCATC	Cloning
pteF_pGREG533_GA_R	CGTGACATAACTAATTACATGACTCGAGTCATGCGTCAGCCCCCTTG	Cloning
pteG_pGREG533_GA_F	CGGACTATGCAGGAGGGAATTCGATGCAGATTCAGCAAGCC	Cloning
pteG_pGREG533_GA_R	CGTGACATAACTAATTACATGACTCGAGTTACTCCTTCATCATCCAGGAGC	Cloning
pteH_pGREG533_GA_F	CGGACTATGCAGGAGGGAATTCGATGAAAATAAACCAAAATC	Cloning
pteH_pGREG533_GA_R	CGTGACATAACTAATTACATGACTCGAGCTAATTTCCTGTAAGATTTGGC	Cloning
pteI_pGREG533_GA_F	CGGACTATGCAGGAGGGAATTCGATGCGTATCGATGGTGTTCGTG	Cloning
pteI_pGREG533_GA_R	CGTGACATAACTAATTACATGACTCGAGTCACACCTTGCCGCGAAAG	Cloning
pteJ_pGREG533_GA_F	CGGACTATGCAGGAGGGAATTCGATGAAAGCCAAATTCAATCTCGCC	Cloning
pteJ_pGREG533_GA_R	CGTGACATAACTAATTACATGACTCGAGTTATTCGGTCAGCAGGCGGTAG	Cloning
pteK_pGREG533_GA_F	CGGACTATGCAGGAGGGAATTCGATGCAGATCCACGCCCTAC	Cloning
pteK_pGREG533_GA_R	CGTGACATAACTAATTACATGACTCGAGTCATCCCAGATAGTTGTTGCTG	Cloning
pteL_pGREG533_GA_F	CGGACTATGCAGGAGGGAATTCGATGAAAACCAGCTCTATCACAGG	Cloning
pteL_pGREG533_GA_R	CGTGACATAACTAATTACATGACTCGAGTTATAACCAACCTTCGGCCAG	Cloning
pteM_pGREG533_GA_F	CGGACTATGCAGGAGGGAATTCGATGCCACCGCCGCCGC	Cloning
pteM_pGREG533_GA_R	CGTGACATAACTAATTACATGACTCGAGTCATAGTACGGATACCTCGATCAGTCG	Cloning
aexT_seq_1_F	GCCGAAGACTCAGGTAGCAG	Sequencing
aexT_seq_2_F	CTACACCAATGGCGAGTACC	Sequencing
aexU_seq_1_F	GAACAACATTGCCGCCGAAC	Sequencing
aexU_seq_2_F	GCTCCATCAACAGTTCCCAC	Sequencing
aopX_seq_1_F	CAGGATGCAATCCAGCACTG	Sequencing
aopO_seq_1_F	CCTCACCGAGACGCTCCATG	Sequencing
aopO_seq_2_F	CGGGATAGTCCACAACGACATC	Sequencing
aopO_seq_3_F	ATCCGCGAGCTCTCCGATAC	Sequencing
aopP_seq_1_F	GCAGCCAACAATAAGCATGTGG	Sequencing
ati2_seq_1_F	AGGCAGTGGCAACGTTAAGG	Sequencing
ati2_seq_2_F	ATCGCTGAAGGTTCTGACGTTATG	Sequencing
aopH_seq_1_F	ACAAGAGCACAGTGCGATTC	Sequencing
aopH_seq_2_F	AGCTGGAGAGTCATCTGCAG	Sequencing
aopS_seq_1_F	CAACTTCTGTTGCCAAAGCC	Sequencing

aopS_seq_2_F	TCGTTCTACAACCCAACCGG	Sequencing
pteA_seq_1_F	CTACCAGGCCCAGCCAAC	Sequencing
pteA_seq_2_F	CCACCTCAAGGAGGTTCTGG	Sequencing
pteA_seq_3_F	CCTTAGTCTGCTGCGCATCG	Sequencing
pteA_seq_4_F	TGCTAGATGGGCTGGCGATG	Sequencing
ateA_seq_1_R	AGCAGAGAGAGGGCTCTTG	
ateA_seq_2_R	ACTCACCCCGAACATCAGG	
pteB_seq_1_F	GATTTCTTTAGCCGCGCCAC	Sequencing
pteC_seq_1_F	TTGTCGCCAAGGTGTTGACC	Sequencing
pteD_seq_1_F	TGTTCTCTCATCGCACCACC	Sequencing
pteD_seq_2_F	TGGAGCCAATGAGCCCAC	Sequencing
pteD.1_seq_1_F	TTAGGATTGGTCTCGAGTGAG	Sequencing
pteE_seq_1_F	CAGACACATGCCCTAGATACGC	Sequencing
pteE_seq_2_F	CCTCACCGATCTTGCACTCTC	Sequencing
pteF_seq_1_F	TGACCAGCGACGGCAAAC	Sequencing
pteG_seq_1_F	GCACACTCTCTGGCTCCTC	Sequencing
pteG_seq_2_F	ACACATTTCGGGCTCATTCC	Sequencing
pteG_seq_3_F	AGTTCCAGGATCTGGAGTCG	Sequencing
pteH_seq_1_F	ACTGCCGCACTTCTGATGAC	Sequencing
pteH_seq_2_F	CCTGAAGGTTGCAATTCTCAGG	Sequencing
pteI_seq_1_F	TGCTAACTGGGGTCCTCAGG	Sequencing
pteJ_seq_1_F	ATGCAGCAAACCAACTTCGC	Sequencing
pteK_seq_1_F	CCGCATATCGCCAGCTTCAC	Sequencing
pteK_seq_2_F	TCCATATTCCCGGCAAGAGC	Sequencing
pteL_seq_1_F	CCCCTCAAGAGATCGCTCTG	Sequencing
pteL_seq_2_F	CATTAGAGGTGCGACAACCG	Sequencing
